# Genome-Wide Gene Expression Analysis in Cancer Cells Reveals 3D Growth to Affect ECM and Processes Associated with Cell Adhesion but Not DNA Repair

**DOI:** 10.1371/journal.pone.0034279

**Published:** 2012-04-11

**Authors:** Oliver Zschenker, Thomas Streichert, Stephanie Hehlgans, Nils Cordes

**Affiliations:** 1 OncoRay – National Center for Radiation Research in Oncology, Medical Faculty Carl Gustav Carus, Dresden University of Technology, Dresden, Germany; 2 Department of Clinical Chemistry/Central Laboratories, University Hospital Hamburg-Eppendorf, Hamburg, Germany; 3 Department of Radiation Oncology, University Hospital and Medical Faculty Carl Gustav Carus, Dresden University of Technology, Dresden, Germany; University Medical Center Hamburg-Eppendorf, Germany

## Abstract

Cell morphology determines cell behavior, signal transduction, protein-protein interaction, and responsiveness to external stimuli. In cancer, these functions profoundly contribute to resistance mechanisms to radio- and chemotherapy. With regard to this aspect, this study compared the genome wide gene expression in exponentially growing cell lines from different tumor entities, lung carcinoma and squamous cell carcinoma, under more physiological three-dimensional (3D) versus monolayer cell culture conditions. Whole genome cDNA microarray analysis was accomplished using the Affymetrix HG U133 Plus 2.0 gene chip. Significance analysis of microarray (SAM) and t-test analysis revealed significant changes in gene expression profiles of 3D relative to 2D cell culture conditions. These changes affected the extracellular matrix and were mainly associated with biological processes like tissue development, cell adhesion, immune system and defense response in contrast to terms related to DNA repair, which lacked significant alterations. Selected genes were verified by semi-quantitative RT-PCR and Western blotting. Additionally, we show that 3D growth mediates a significant increase in tumor cell radio- and chemoresistance relative to 2D. Our findings show significant gene expression differences between 3D and 2D cell culture systems and indicate that cellular responsiveness to external stress such as ionizing radiation and chemotherapeutics is essentially influenced by differential expression of genes involved in the regulation of integrin signaling, cell shape and cell-cell contact.

## Introduction

The microenvironment is a fundamental regulator of cell behavior [1], [2], [3]. A large body of work has shown how interactions of cells with the extracellular matrix (ECM), as one of the key components of the microenvironment, contribute to the regulation of critical cell functions such as cell shape/architecture, survival, proliferation, and differentiation [2], [4], [5], [6], [7], [8], [9]. Cell-ECM interactions also control gene expression and chromatin organization in a growth and ECM-dependent manner [10], [11]. Recent emerging findings show that especially the growth conditions play an essential role for the cellular responsiveness to external stimuli. This became evident in three-dimensional (3D) ex vivo cell cultures grown in ECM and in spheroid models [4], [11], [12], [13], [14], [15], [16], [17]. Importantly, these 3D cell culture models better mimic a physiological microenvironment than conventional uncoated or ECM-precoated cell culture plastic [15], [18].

In addition to the use of 3D cell culture models in tissue engineering [19], [20], [21] and studies on embryonic development and physiology [18], 3D cell cultures are increasingly employed in cancer research [7], [8], [9], [12], [16], [22]. In the vast majority of cases, tumor cell lines of different origin show an enhanced resistance to radio- and chemotherapy in a 3D environment indicative by increased clonogenicity and decreased apoptosis [12], [13], [14], [16], [17], [23], [24], [25], [26], [27]. Apart from a significant impact of integrin-mediated cell-ECM interactions [28], a complex interplay of biochemical signaling pathways and biophysical/mechanotransduction-related factors is thought to confer this enhanced tumor cell resistance whose underlying mechanisms remain to be determined both on the gene and on the protein level [2].

With regard to gene expression, great efforts have been undertaken to identify specific diagnostic, prognostic and therapy-monitoring gene expression patterns in biopsies of various human malignancies [2], [5], [6], [29], [30], [31]. Intriguingly, some of these studies demonstrated strong overlap between in vivo and 3D but not 2D cell culture data sets, which finally enabled the identification of gene signatures predictive for overall survival of cancer patients. It remains to be clarified whether changes in gene expression under 3D versus 2D growth conditions can explain or provide hints at certain stress or DNA repair pathways involved in the enhanced radio- or chemoresistance of 3D grown tumor cells. If so, targeted therapeutic approaches against key tumor promoters could be optimized.

To address this question, this study compared basal gene expression of two human cancer cell lines of different origin and with varying genetic background in a 3D ECM scaffold or under conventional 2D monolayer conditions with respect to their behavior upon radiation and chemotherapy.

## Materials and Methods

### Cell lines, culture conditions, and cell doubling times

Lung tumor cell line A549 was obtained from ATCC (Manassas, USA). The squamous cell carcinoma cell line UT-SCC15 was a kind gift from R. Grenman (Turku University Central Hospital, Finland). For conventional 2D cell culture, cells were cultured in Dulbecco's Modified Eagle Medium (DMEM; PAA, Cölbe, Germany) containing glutamax-I (L-alanyl-L-glutamine) supplemented with 10% fetal calf serum (FCS; PAA) and 1% non-essential amino acids (NEAA; PAA) at 37°C in a humidified atmosphere containing 7% CO_2_. For 3D cell culture, cells were plated into a mixture of 0.5 mg/ml laminin-rich extracellular matrix (Matrigel; BD, Heidelberg, Germany) and complete DMEM medium upon a layer of agarose (Sigma, Taufkirchen, Germany) in a 24-well cell culture dish (BD) as published [12], [13], [14], [16].

Doubling time of cells growing as monolayers in cell culture flasks were counted according to standard protocols. Briefly, single cells were plated in 2D or 3D and trypsinized and transferred to 10 ml of medium containing FCS. After gently mixing in medium, 10 µl of the cell solution was pipetted onto a Neubauer counting chamber using an appropriate dilution. Cells in 4 squares were counted microscopically (Zeiss, Jena, Germany) and the number of cells was calculated as previously described [32]. For cells growing in a 3D Matrigel environment, cells were isolated as previously described [23], [33] using a Neubauer counting chamber.

### 3D and 2D colony forming assays

Clonogenic survival under 3D conditions was tested as previously published [15], [16], [17], [34]. Briefly, single cells were plated in 0.5 mg/ml Matrigel supplemented with DMEM/10% FCS/1% NEAA. After 24 h, single cells were irradiated (0–6 Gy) or treated with increasing Cisplatin concentrations (0.1, 1, 5, 10 µM; for 24 h; Neocorp). Cisplatin was removed by 3- (2D) or 15-times (3D) washing with DMEM/10% FCS/1% NEAA. Then, cells were allowed to grow to colonies/cell clusters. At 8 (A549) or 11 days (UT-SCC15) after plating, grown colonies/cell clusters (>50 cells) were microscopically counted. 2D cell survival was accomplished as published [23]. After 8 days (A549) or 11 days (UT-SCC15), cells were stained with Coomassie blue (Merck, Darmstadt, Germany) and colonies (>50 cells) were counted. Plating efficiencies were calculated as follows: numbers of colonies formed/numbers of cells plated. Surviving fractions were calculated as follows: numbers of colonies formed/(numbers of cells plated (irradiated/Cisplatin)×plating efficiency (unirradiated/untreated)). Each point on survival curves represents the mean surviving fraction from three independent experiments.

### Irradiation of cell cultures

Irradiation was delivered at room temperature using single doses (0–6 Gy) of 200 kV X-rays (Yxlon Y.TU 320; Yxlon, Copenhagen, Denmark) filtered with 0.5 mm copper. The dose-rate was approximately 1.3 Gy/min at 20 mA. The absorbed dose was evaluated using a Duplex dosimeter (PTW, Freiburg, Germany).

### cDNA microarray

Gene expression profile was measured with RNA extracted from 5×10^6^ cells grown in 3D or 2D. Total RNA was prepared using NucleoSpin RNA II Kit according to the manufacturer's instructions (Macherey-Nagel, Düren, Germany). Procedures for cDNA synthesis, labeling and hybridization were carried out according to the manufacturer's protocol (Affymetrix) and as published [35]. All experiments were performed using Affymetrix human genome gene chip HG U133 Plus 2.0. First strand cDNA synthesis with 90 ng of total RNA, synthesis of biotin-labeled cRNA and clean up was carried out using the 3′ IVT Express Kit (Affymetrix). For hybridization, 15 µg of fragmented cRNA were incubated with the chip in 200 µl of hybridization solution in Hybridization Oven 640 (Affymetrix) at 45°C for 16 hours. GeneChips were then washed and stained with the Affymetrix Fluidics Station 450 according to the GeneChip Expression Wash, Stain and Scan Manual using the GeneChip Hybridization, Wash and Stain Kit (Affymetrix). Microarrays were scanned with the Affymetrix GeneChip Scanner 7G, and the signals were processed using GCOS (v.1.4; Affymetrix). To compare samples and experiments the RMA algorithm was used (Expression Console, v.1.1), further analysis was carried out with TIGR MeV (v.4.6.2). Three replicates were used for microarray analysis and statistics. For SAM analysis, a Signal Log Ratio of 0.8 (−0.8) was used as a threshold which equals a fold change of 1.6 and −1.6, respectively. Gene expression data are available at GEO Accession No. GSE17347. For gene list, functional enrichment we used the ToppGeneSuite [36]. Pathways and other functional groupings of genes were evaluated for differential regulation using the visualization tool GenMAPP (Ver. 2.1) as described previously [37].

SAM requires the input of a “delta” value. The “delta” value defines the threshold of the number of false positive genes in the validated dataset. To identify a list of potentially significant genes, we calculated the false discovery rate (FDR). The estimated FDR (the median number of falsely significant genes) for each given “delta” (“delta” value) was determined according to Tusher et al. [38].

### Semi-quantitative RT-PCR

For validating microarray data by PCR, total RNA isolated for gene expression profiling was used. cDNA was prepared with SuperScript™ III Reverse Transcriptase kit according to the instructions of the manufacturer (Invitrogen, Karslruhe, Germany). Briefly, cDNA was synthesized in a 20 µl volume containing 1 µg of DNase-treated total RNA, 1 µl of oligo(dt)_20_ (50 µM), and 1 µl 10 mM dNTP Mix, 4 µl of 5× First Strand buffer, 1 µl of 0.1 M DTT, 1 µl of RNase OUT, and 1 µl of SuperScript III (200 U/µl). RNA, dNTPs and oligo(dt) primer were mixed first, heated to 65°C for 5 min, and placed on ice until addition of the remaining reaction components. Then, the reaction mixture was incubated at 55°C for 45 minutes and terminated by heat-inactivation at 70°C for 15 minutes. An identical reaction without the reverse transcriptase was performed to verify the absence of genomic DNA. Semi-quantitative RT-PCR was performed for the genes TXNIP, DUSP6, CEACAM1, NPC1 and BCL2A1 using primers listed in Table 1 (Eurofins MWG Operon, Ebersberg, Germany), 2 µl of cDNA and HotStar Taq polymerase (Qiagen, Hilden, Germany) according to standard PCR protocols. Forward primers for all genes were chosen by searching the original oligonucleotide sequence of the corresponding gene identification number of the Affymetrix gene chip on the Affymetrix web site (http://www.affymetrix.com/analysis/index.affx). The reverse primers were designed based on the gene sequence provided on the Affymetrix web page. Annealing temperatures of 50°C were used for TXNIP, DUSP6 and BCL2A1 or 55°C for CEACAM1 and NPC1. For normalization, a β-actin fragment of 540 bp was amplified concurrently using primers listed in Table 1 [39]. The results of three independent experiments were analyzed using 1% agarose (Carl Roth, Karlsruhe, Germany) gels and densitometric analysis with Image J® software (National Institutes of Health, Bethesda, USA) after staining gels with ethidium bromide (Carl Roth).

**Table 1:** Primers used for RT-PCR and quantitative real-time PCR including calculated sizes of PCR products.

Gene	Primer forward (5′→3′)	Primer reverse (5′→3′)	Size [bp]
**TXNIP**	GAAGCAGCTTTACCTACTTGTTTCT	AAACTATCGAAAAGGCCTCAATTTT	292
**DUSP6**	AATTGTGCTCTTTTCTAATCCAAA	AAAGTGAGCCCATGATTTGGTGTCTTT	250
**CEACAM1**	GACAGGCAAATGTACTTCTCACCCA	AAAGAGGTACCTGAGTATAGAGAAC	162
**NPC1**	TAAGCCATCCCACAAGTTCTATACC	GGAGACCAAGCTCTAATGAGGCC	182
**BCL2A1**	AAAATGTTGCGTTCTCAGTCCAAA	ATTTAGGTTCAAACTTCTTTACAAA	342
**β-actin**	GTGGGGCGCCCCAGGCACCA	CTCCTTAATGTCACGCACGATTTC	540

### Total protein extracts and Western Blotting

Total protein extracts from 4-day 3D and 2D cell cultures were isolated as previously described [13]. In brief, cells were treated with modified RIPA buffer (50 mM Tris-HCl (Carl Roth), pH = 7.4), 1% Nonidet-P40 (Sigma), 0.25% sodium deoxycholate (AppliChem, Darmstadt, Germany), 150 mM NaCl (VWR International, Darmstadt, Germany), 1 mM ethylenediaminetetraacetic acid (Merck), complete protease inhibitor cocktail (Roche, Mannheim, Germany), 1 mM NaVO_4_ (AppliChem), 2 mM NaF (AppliChem)). Samples were stored at −80°C. Total protein amount was measured using the bicinchoninic acid assay (Pierce, Bonn, Germany). After sodium dodecyl sulfate polyacrylamide gel electrophoresis and transfer of proteins onto nitrocellulose membranes (Schleicher & Schüll, Dassel, Germany), proteins were detected with the following antibodies: anti-Fibronectin (BD, Heidelberg, Germany), anti-CTGF, anti-ErbB3 (Santa Cruz, Heidelberg, Germany), anti-BCL2A1 (LifeSpan Biosciences, Seattle, USA) and anti-β-actin (Sigma); horseradish peroxidase-conjugated donkey anti-rabbit, donkey anti-goat and sheep anti-mouse (Amersham, Freiburg, Germany) secondary antibodies. SuperSignal® West Dura Extended Duration Substrate (Pierce, Bonn, Germany) and Amersham Hyperfilm ECL (GE, Munich, Germany) were used for detection. Densitometry was performed using Image J® software. Three independent experiments have been performed.

## Results

### 3D and 2D cell growth characteristics and cell survival after radio- or chemotherapy

**Figure 1:**
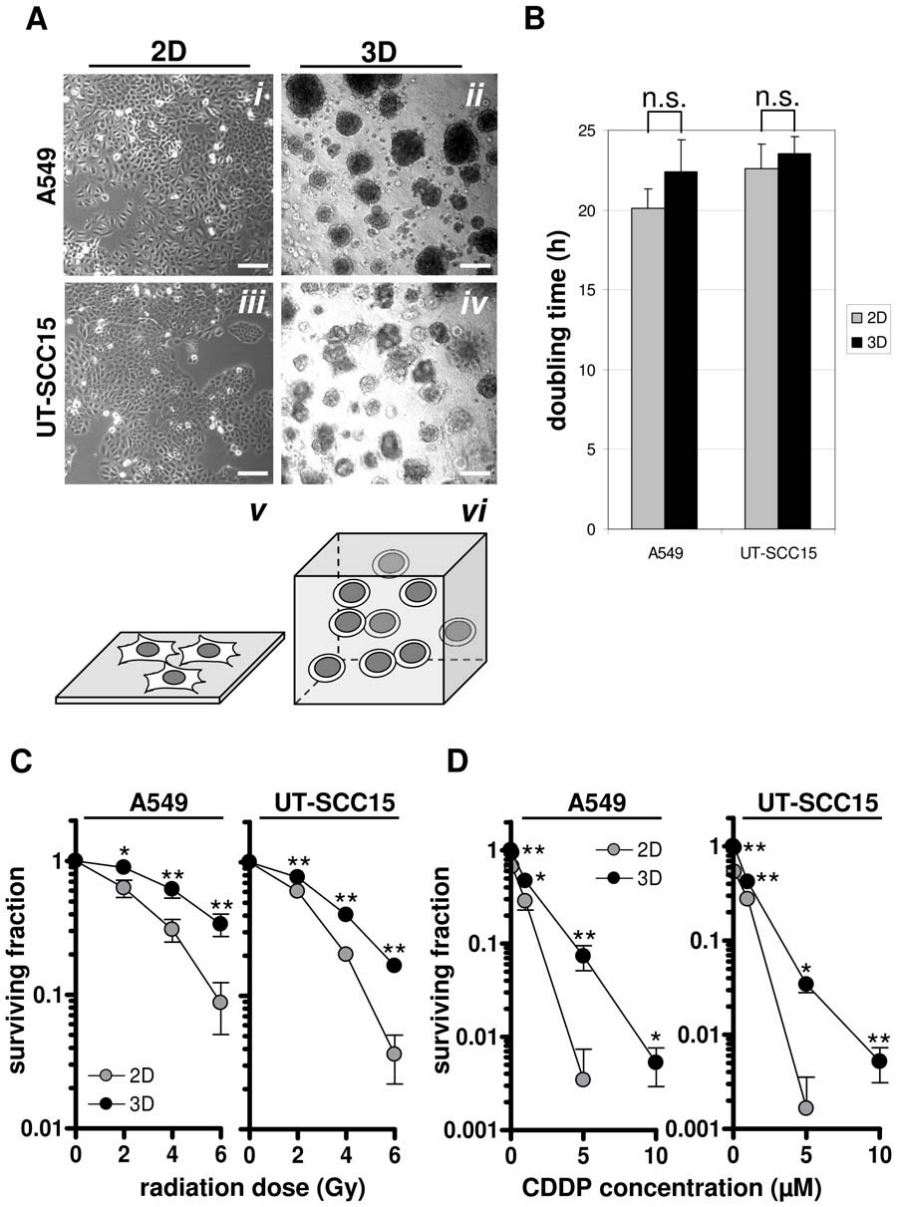
Cell morphology, doubling times and cellular radiosensitivity of A549 and UT-SCC15 cells under 2D or 3D growth conditions. (A) Representative pictures and schematic of 2D (*i*, *iii*) and 3D (*ii, iv*) cell growth as found at day 4 immediately before RNA isolation. Bars, 50 µm. (B) Doubling times of 2D and 3D cell cultures at day 4 after plating. Results show mean ± SD (n = 3). n.s., not significant. Clonogenic survival of 2D or 3D grown cells exposed to increasing X-ray doses (2, 4 or 6 Gy) (C) or Cisplatin concentrations (0.1, 1, 5, 10 µM) (D) applied 24 h after plating. Cisplatin was removed after 24 h by 3- (2D) or 15-times (3D) washing with DMEM/10% FCS/1% NEAA. Results show mean ± SD (n = 3; t-test). * P<0.05; ** P<0.01. CDDP, Cisplatin. Bar, 200 µm.

Cell shape and morphology were different between 3D and 2D cell growth conditions (Fig. 1A). Four days after plating, cells were exponentially growing with similar doubling times (Fig. 1B). This similarity in cell doubling times provided comparable experimental conditions for the whole genome gene expression analysis. Importantly, previous experiments evaluating the radiation survival of A549 and UT-SCC15 cells were repeated thus forming the rationale for the presented study to link growth-dependent radio- and chemoresistance with growth-dependent gene expression modification [11]. Under 3D conditions, both cell lines showed significant higher clonogenic survival upon exposure to increasing single doses of X-rays or increasing concentrations of Cisplatin as compared to 2D (Fig. 1C and D). These data suggest a connection between growth conditions and cell morphology and cellular radio- and chemoresistance.

### Whole genome gene expression analysis of A549 and UT-SCC15 cultured in 3D or 2D

**Figure 2:**
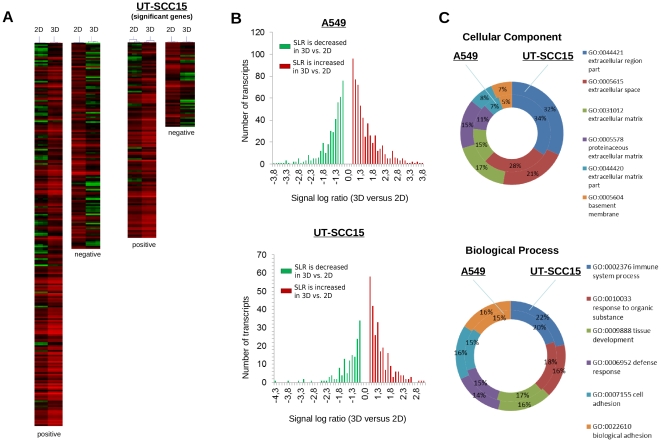
Clusters and RNA ratios of differentially expressed genes in 3D and 2D cell cultures of A549 and UT-SCC15 cells. (A) Hierarchical clusters of genes of 2D and 3D cell cultures at day 4 after plating. Red indicates genes expressed above average; green indicates genes expressed below average and black indicates average expression after Significance Analysis of Microarrays (SAM). “Positive” indicates genes upregulated in 3D versus 2D. “Negative” indicates genes downregulated in 3D versus 2D. (B) Plot of number of transcripts against signal log ratios from 3D versus 2D cell cultures of A549 and UT-SCC15 cells. (C) Gene Ontology-dependent plotting of absolute numbers and percentage of significantly modified genes in 3D versus 2D cell cultures according to SAM analysis.

This genome wide gene expression analysis was performed under untreated conditions and intended to identify specific gene expression patterns of single genes or functionally associated gene groups that can be linked to tumor cell radio- and chemoresistance. From gene profiling, hierarchical clustering and statistical analysis using SAM, it was obvious that growth conditions affect gene expression patterns (Fig. 2A and B). The estimated false discovery rate (FDR) was 0 down to the set “delta” (“delta” = 2.873 in A549; “delta” = 2.445 in UTSCC15). A higher number of genes were differentially expressed in A549 (376 transcripts) than in UT-SCC15 (178 transcripts) cells (Fig. 2A, Table 2; Table S1 and S2). In 3D, A549 and UT-SCC15 cells showed more genes up-regulated than down-regulated as compared to 2D (Fig. 2A and B).

**Table 2:** z-scores calculated for selected GO terms and pathways^a^.

	A549		UT-SCC15	
	z-score	No. of genes changed	z-score	No. of genes changed
***Pathways***				
Regulation of cell size	**4.487**	15	1.853	5
Cell-cell signaling	−0.06	11	**2.324**	10
TGF-beta signaling	**3.211**	7	**2.019**	3
Nuclear receptors	**4.24**	7	0.316	1
DNA repair	−1.738	2	−1.697	0
***Cellular Component***				
GO:0044421 extracellular region part	/	/	/	/
GO:0005615 extracellular space	**9.465**	50	**5.714**	21
GO:0031012 extracellular matrix	**5.486**	24	**6.897**	17
GO:0005578 proteinaceous extracellular matrix	**5.621**	24	**7.025**	17
GO:0044420 extracellular matrix part	/	/	/	/
GO:0005604 basement membrane	**5.514**	8	**5.42**	5
***Biological Process***				
GO:0002376 immune system process	/	/	/	/
GO:0010033 response to organic substance	−0.642	0	**1.96**	1
GO:0009888 tissue development	0.734	7	**5.635**	11
GO:0006952 defense response	**2.581**	38	**5.072**	28
GO:0007155 cell adhesion	**4.532**	36	**5.147**	22
GO:0022610 biological adhesion	/	/	/	/

a z-scores were calculated according to the GenMAPP manual based on the genes identified by t-test. A z-score value of −1.96 or 1.96 corresponds to a p-value of 0.05. The higher the absolute value of the z-score the more significant is the enrichment of changed genes in the scored pathway. Significant z-scores are high-lighted in bold letters.

According to SAM, A549 cells had 242 transcripts up- and 134 down-regulated while UT-SCC15 cells had 125 transcripts up- and 53 transcripts down-regulated (Fig. 2A and B, Table S1 and S2). Intriguingly, these two different cell lines showed an overlap of cellular components and biological functions to be affected under 3D growth conditions with regard to gene ontology (Fig. 2C, Tables S3 and S4). UT-SCC15 cells, in general, demonstrated less genes modified by 3D growth as compared to A549 cells (Fig. 2A, Table S2). From a one-to-one gene expression comparison, it became obvious that the modifications occur in different genes within the same groups of cellular components or biological functions in a cell line-dependent manner. On top of SAM, t-test analysis was performed in which 856 genes were up-regulated and 721 down-regulated in 3D A549 cell culture relative to 2D (to note: SLR threshold +/− 0.8). In UT-SCC15 cells, 429 genes were up-regulated and 277 down-regulated in 3D as compared to 2D. The overlap of differentially expressed genes upon SAM and t-test for the 3D-to-2D comparison revealed 238 transcripts up-regulated and 128 transcripts down-regulated in A549 cells and 119 transcripts up-regulated and 52 transcripts down-regulated in UT-SCC15 cells (Table S5 and S6).These findings indicate that processes associated with cell adhesion, biological adhesion, immune system responses, defense responses, tissue development and response to organic substance are predominantly regulated via differential gene expression when cells are transferred from a 2D to a 3D matrix-based microenvironment. Without pronounced expression changes of genes involved in DNA repair, it can be speculated that radio- and chemoresistance of 3D grown cells results from particular, yet unidentified, changes of cell architecture and hereby induced modifications of the cellular interactome in terms of signal transduction and protein-protein interactions.

### Validation of microarray data by PCR and Western Blotting

For microarray data validation, selected genes and proteins were examined using semi-quantitative RT-PCR (thioredoxin interacting protein (TXNIP), dual specificity phosphatase 6 (DUSP6), carcinoembryonic antigen-related cell adhesion molecule 1 (CEACAM1), Niemann-Pick disease, type C1 (NPC1) and BCL2-related protein A1 (BCL2A1)) and Western Blotting (Fibronectin (FN1), connective tissue growth factor (CTGF), v-erb-b2 erythroblastic leukemia viral oncogene homolog 3 (ErbB3) and BCL2A1), respectively.

**Figure 3:**
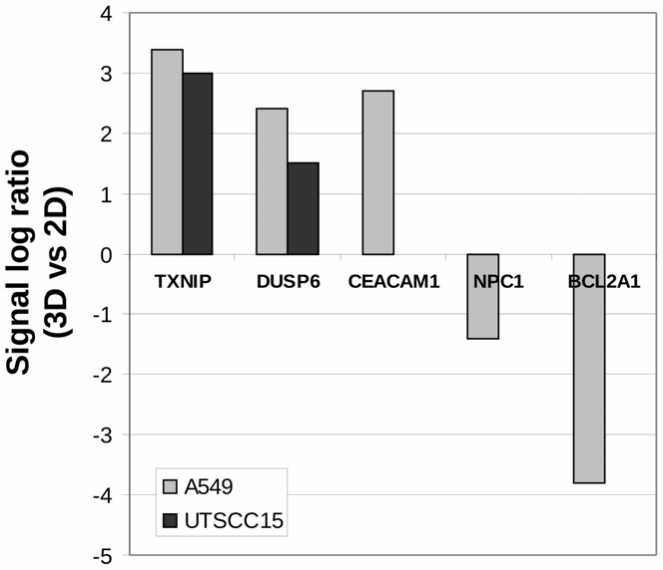
Signal log ratios of selected genes from DNA microarray-based analysis subjected to semi-quantitative RT-PCR verification. Expression of the genes TXNIP1, DUSP6, CEACAM1, NPC1 and BCL2A1 is delineated comparatively from 3D versus 2D cell cultures of A549 and UT-SCC15 cells.

**Figure 4:**
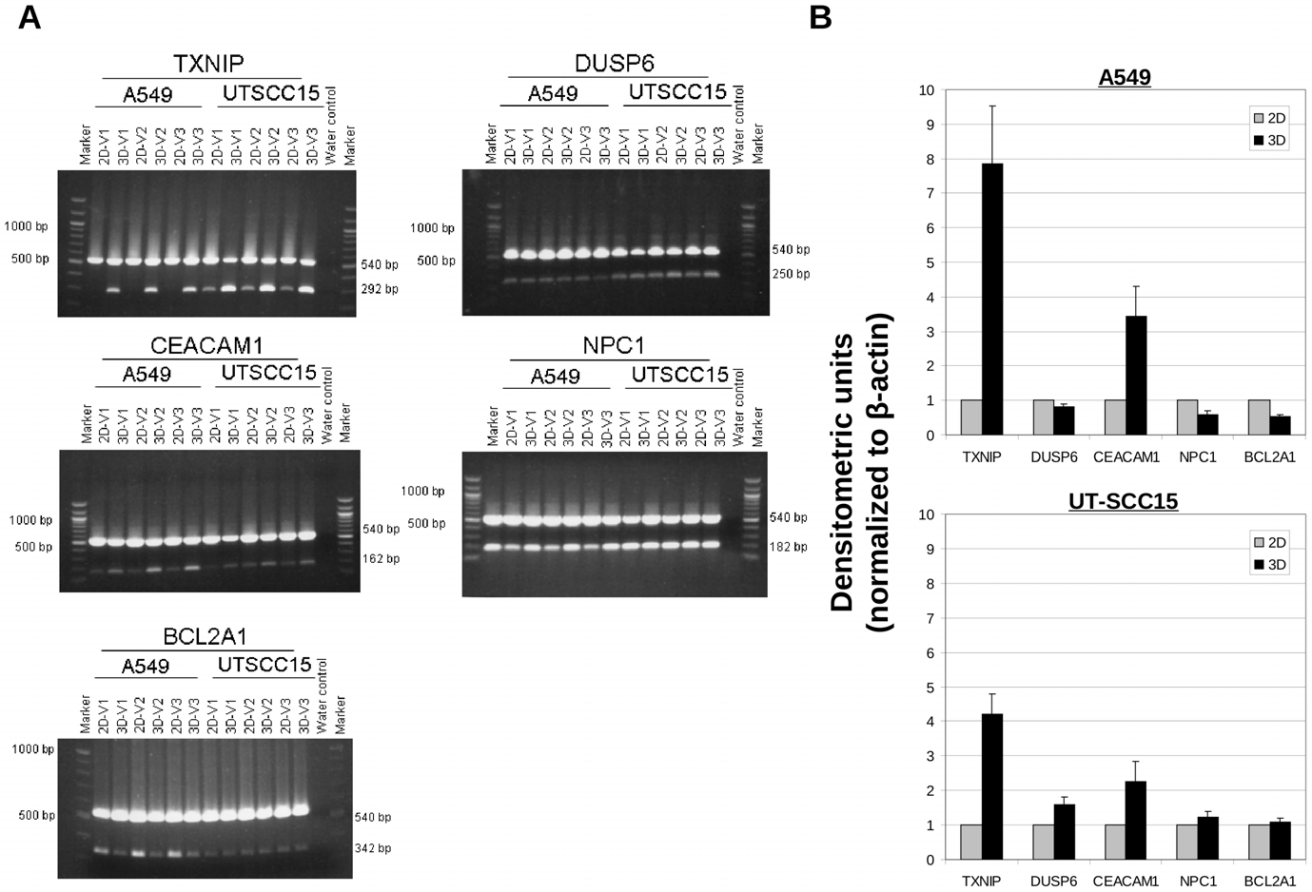
Validation of microarray gene expression data by semi-quantitative RT-PCR. (A) Semi-quantitative RT-PCR was performed as described under Materials and Methods. Shown are 1% agarose gels with RT-PCR fragments of TXNIP, DUSP6, CEACAM1, NPC1 and BCL2A1 mRNAs isolated from A549 or UT-SCC15 cells. Samples were amplified from cDNA generated by reverse transcription of total RNA of 4-day old 3D and 2D cell cultures. All experiments (V1, V2, V3) are exhibited. β-actin expression served as loading control (540 bp). (B) Graphs display results from densitometric analysis of indicated mRNA expression after normalization to β-actin. Results show mean ± SD (n = 3).

The signal log ratios of selected genes from 3D or 2D cell cultures are displayed in Figure 3. TXNIP and DUSP6 present genes increasingly expressed in both cell lines when cultured in 3D relative to 2D (Fig. 3). CEACAM1 induction and NCP1 and BCL2A1 repression were only observed in 3D A549 cell cultures (Fig. 3). Semi-quantitative RT-PCR on RNA samples used for microarray analysis confirmed induced TXNIP expression in 3D while increased DUSP6 expression could only be confirmed in UT-SCC15 cells (Fig. 4A and B). Enhanced CEACAM1 mRNA levels were detectable in both 3D A549 and 3D UT-SCC15 cell cultures, which was confirmatory for A549 and borderline for UT-SCC15 cells with regard to DNA microarray data (Fig. 4A and B). The genes NPC1 and BCL2A1 showed reduced or stable levels in the genome wide analysis in A549 or UT-SCC15 cells, respectively; results fully confirmed by semi-quantitative RT-PCR (Fig. 4A and B).

**Figure 5:**
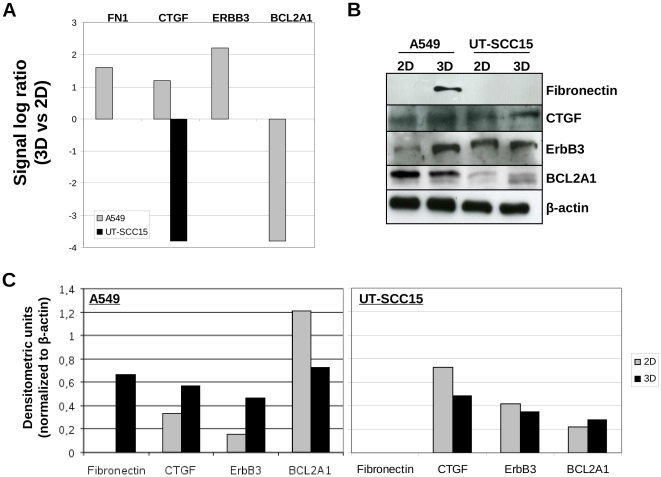
Validation of microarray gene expression data on protein level. (A) Signal log ratios of selected genes (FN1, CTGF, ERBB3 and BCL2A1) from DNA microarray-based analysis subjected to Western blot analysis. (B) Whole cell lysates were prepared from 3D and 2D cell cultures on day 4 after plating. SDS-PAGE and Western blot analysis were performed as described under Material and Methods. Representative images show expression of Fibronectin (240 kDa), CTGF (38 kDa), ErbB3 (180 kDa) and BCL2A1 (20 kDa). β-actin served as loading control. (C) Densitometric units of protein bands shown in ‘B’ are plotted upon normalization to β-actin.

Next, a different set of genes (FN1, CTGF, ERBB3, BCL2A1) was checked on the protein level by Western blotting. The signal log ratios of these genes generated by the DNA microarray were: FN1 = 1.6/n.c. (A549/UT-SCC15); CTGF = 1.2/−3.8 (A549/UT-SCC15); ERBB3 = 2.2/n.c. (A549/UT-SCC15); BCL2A1 = −3.8/n.c. (A549/UT-SCC15) (Fig. 5A, Table S1 and S2). In 3D A549 cell cultures, protein expression analysis confirmed the DNA microarray-based up-regulation of Fibronectin, CTGF and ERBB3 as well as the down-regulation of BCL2A1 (Fig. 5B and C). 3D UT-SCC 15 cell cultures showed no changes of examined proteins, including CTGF, which demonstrated strong repression according to our microarray-based analysis (Fig. 5B and C). In most instances, these data showed similar results between whole genome wide gene expression analysis using microarray format and RT-PCR or Western blotting.

## Discussion

Loss of functional and phenotypic characteristics occur when cells are cultured ex vivo in a 2D microenvironment [1], [4], [18], [20]. This can be prevented by culturing cells in physiological 3D ECM scaffolds shown for various endpoints such as protein expression, morphology, and prediction of gene signatures for clinical outcome [2], [5], [6], [29], [30]. Considering these points, we focused on the mechanisms modulating tumor cell sensitivity to radio- and chemotherapy. To assess the impact of basal gene expression profiles on the more physiological 3D versus the artificial 2D cell culture models, this study was performed on human epithelial cancer cell lines originating from two of the most frequent cancers worldwide, i.e. lung (A549) and head and neck squamous cell carcinoma (UT-SCC15) [40]. Selected from various cell line models tested in our laboratory, these two cancer cell lines vary in their origin and genetic background and are prime examples showing the prosurvival effects of growth in a 3D ECM scaffold in comparison to conventionally used culture plastic [11], [12], [13], [14]. We show significant changes in gene expression profiles of 3D versus 2D cell cultures. While genes involved in DNA repair pathways stayed unmodified, the majority of altered genes in both cell lines were associated with biological functions like tissue development, cell adhesion, and defense response. Validation of microarray data was performed on selected genes on mRNA and protein level.

3D cell cultures are increasingly employed in cancer research as well as tissue engineering, developmental and cell biology. For therapy development, cell responsiveness is the key issue and dramatically different in a 3D physiological environment as compared to Petri dish conditions. With regard to clonogenic cell survival after exposure to X-rays or the widely applied chemotherapeutic drug Cisplatin, both A549 and UT-SCC15 cells show increased survival rates in 3D relative to 2D. As these are only two examples out of several [12], [13], [14], [15], [23], [24], the paradigms “cell adhesion mediated radioresistance” and “cell adhesion mediated drug resistance” mainly examined in conventional ECM-coated cell culture dishes have to be re-evaluated by taking severe modifications in e.g. signal transduction, DNA repair processes, etc into account when cells grow in 3D. Importantly, differences in parameters such as hypoxia, proliferation and radiation dosimetry that are well known as essential determinants of cellular radiosensitivity could be excluded in a previous in-depth comparative analysis between 3D and 2D cell culture conditions [11]. Thus, the 3D ECM-based cell culture model used here is a reasonable and feasible method for investigations of tumor cell radio- and chemosensitivity and the underlying mechanisms.

Despite our knowledge about severe alterations of gene expression patterns by separating cells from tissues for ex vivo 2D cell cultures [1], [41], [42], these findings have been widely neglected. In 2D, both the number of differentially expressed genes and the gene expression levels in cell lines from tumor and normal tissues were diminished by up to 70% and had a fluctuation range of about 1.5-fold as compared to the originating tissues, respectively [43], [44]. Comparing our microarray analysis with the work of others, we found similar gene expression profiles. For example, both A549 and UT-SCC15 showed distinctive basal patterns of genes encoding for proteins with functions in the modulation of the ECM like Laminin, Fibronectin, Collagen, a finding similarly observed in a basal gene expression analysis of 60 cancer cell lines and their corresponding cancer tissues and in a study done on melanoma [30], [42]. These data further support our notion that 3D cell cultures are more physiological and tissue-like than 2D. Despite similar growth rates of our tested human tumor cell lines, cellular differentiation processes associated with modifications in e.g. ECM proteins could pronouncedly influence the responsiveness of tumor cells to various therapies.

Of upmost importance for us was the finding that 3D cell cultures differentially express genes involved in the ‘extracellular matrix’ and ‘cell adhesion’ as well as ‘defense response’ but not in ‘DNA repair’. As a modulation of genes involved in the regulation of cell size and adhesion is not astonishing when cells are transferred from monolayer to a 3D environment, these findings strongly suggest that the level of expression of particular genes associated with DNA repair is not necessarily linked functionally to an observed cell behavior. In our case, irradiated or Cisplatin-treated A549 and UT-SCC15 showed a higher clonogenic survival under 3D growth conditions as compared to 2D; however, a corresponding increase in the expression of e.g. DNA repair genes such as PRKDC (protein kinase, DNA-activated, catalytic polypeptide), ATM (ataxia telangiectasia mutated) or MDC1 (mediator of DNA-damage checkpoint 1) was absent. Conclusively and in contrast to study assessing the prediction of radiotherapy and chemotherapy responsiveness, it may be speculated that the major determinant of this improved survival is likely to be the protein interactome and not the transcriptome. Concerning transfer of data from bench-to-bedside for therapy, it should be emphasized that data generated in tumor samples with conserved phenotypes, including gene expression profiles, is likely to be more relevant than 2D data and can be used to identify essential signaling hubs for target therapy.

In summary, the presented data clearly demonstrate that growth conditions have profound impact on gene expression. Since 3D cell cultures reflect physiological growth conditions and confer therapy resistance to cancer cells, these findings suggest that, on the transcriptome level, cell shape and cell-cell contact are two of the major determinants of cellular responsiveness to external stress. This notion can be propagated to a more realistic, higher complexity in which structural and communicative changes in the proteome add substantial cues for the regulation of cellular behavior, in particular therapy resistance. Future studies in optimized cell culture models such as the 3D ECM model are warranted to address the transcriptomic and proteomic mechanisms of tumor cell biology, cancer therapy responsiveness and evaluation of novel potential cancer targets.

## Supporting Information

Table S1 **Gene expression analysis in 3D versus 2D A549 cell cultures.**
(DOC)

Table S2 **Gene expression analysis in 3D versus 2D UT-SCC15 cell cultures.**
(DOC)

Table S3 **Gene Ontology Analysis, Cellular Component. The overlap is marked in italic/bold letters.**
(DOCX)

Table S4 **Gene Ontology Analysis, Biological Process. The overlap is marked in italic/bold letters.**
(DOCX)

Table S5 **Gene expression analysis in 3D versus 2D A549 cell cultures. Overlap T-Test/SAM.**
(DOCX)

Table S6 **Gene expression analysis in 3D versus 2D UT-SCC15 cell cultures. Overlap T-Test/SAM.**
(DOCX)
