# Supplementary material for: Genome-Wide Gene Expression Analysis in Cancer Cells Reveals 3D Growth to Affect ECM and Processes Associated with Cell Adhesion but Not DNA Repair
Source: PLoS One. 2012 Apr 11;7(4):e34279. doi: 10.1371/journal.pone.0034279 (PMC3324525; doi:10.1371/journal.pone.0034279)
Supplement: Table S1 — Gene expression analysis in 3D versus 2D A549 cell cultures. (DOC) [file pone.0034279.s001.doc]

| **Table S1. Gene expression analysis in 3D versus 2D A549 cell cultures.^a^** | | | |  |  |
| --- | --- | --- | --- | --- | --- |
|  |  |  |  |  |  |
| Common name | Affymetrix-ID | Genbank | Description | Fold change | Signal log ratio |
| **Genes upregulated in 3D versus 2D** | | |  |  |  |
| FCGBP | 203240_at | NM_003890 | Fc fragment of IgG binding protein | 13.59 | 3.77 |
| MUC5AC | 214303_x_at | AW192795 | mucin 5AC, oligomeric mucus/gel-forming | 13.13 | 3.69 |
| MUC5AC | 214385_s_at | AI521646 | mucin 5AC, oligomeric mucus/gel-forming | 12.27 | 3.62 |
| MUC5B | 213432_at | AI697108 | mucin 5B, oligomeric mucus/gel-forming | 11.13 | 3.46 |
| FGA | 205649_s_at | NM_000508 | fibrinogen alpha chain | 10.00 | 3.31 |
| FGA | 205650_s_at | NM_021871 | fibrinogen alpha chain | 6.87 | 2.77 |
| TXNIP | 201010_s_at | NM_006472 | thioredoxin interacting protein | 9.87 | 3.35 |
| HAL | 206643_at | NM_002108 | histidine ammonia-lyase | 9.22 | 3.21 |
| CHGB | 204260_at | NM_001819 | chromogranin B (secretogranin 1) | 8.87 | 3.15 |
| TF | 214063_s_at | AI073407 | transferrin | 8.51 | 3.10 |
| TF | 203400_s_at | NM_001063 | transferrin | 7.20 | 2.86 |
| AGTR1 | 205357_s_at | NM_000685 | angiotensin II receptor, type 1 | 8.35 | 3.06 |
| AGTR1 | 208016_s_at | NM_004835 | angiotensin II receptor, type 1 | 7.07 | 2.83 |
| ADH6 | 214261_s_at | H71135 | alcohol dehydrogenase 6 (class V) | 8.33 | 3.04 |
| CP | 204846_at | NM_000096 | ceruloplasmin (ferroxidase) | 8.01 | 3.01 |
| CP | 227253_at | AI922198 | ceruloplasmin (ferroxidase) | 6.56 | 2.72 |
| CP | 1558034_s_at | AL556703 | ceruloplasmin (ferroxidase) | 6.31 | 2.64 |
| CP | 228143_at | AI684991 | ceruloplasmin (ferroxidase) | 5.67 | 2.51 |
| SERPINA1 | 202833_s_at | NM_000295 | serpin peptidase inhibitor, clade A (alpha-1 antiproteinase, antitrypsin), member 1 | 7.96 | 3.00 |
| SERPINA1 | 211429_s_at | AF119873 | serpin peptidase inhibitor, clade A (alpha-1 antiproteinase, antitrypsin), member 1 | 6.88 | 2.79 |
| C13orf15 | 218723_s_at | NM_014059 | chromosome 13 open reading frame 15 | 7.95 | 2.99 |
| FGG | 219612_s_at | NM_000509 | fibrinogen gamma chain | 7.95 | 2.99 |
| AGR2 | 228969_at | AI922323 | anterior gradient homolog 2 (Xenopus laevis) | 7.54 | 2.91 |
| AGR2 | 209173_at | AF088867 | anterior gradient homolog 2 (Xenopus laevis) | 2.42 | 1.27 |
| AGR3 | 228241_at | AI827789 | anterior gradient homolog 3 (Xenopus laevis) | 7.41 | 2.89 |
| FLJ31568 | 222274_at | AW975050 | FLJ31568 protein | 7.30 | 2.76 |
| FOSB | 202768_at | NM_006732 | FBJ murine osteosarcoma viral oncogene homolog B | 7.17 | 2.82 |
| KLF2 | 219371_s_at | NM_016270 | Kruppel-like factor 2 (lung) | 6.78 | 2.75 |
| CYP3A5 | 214235_at | X90579 | cytochrome P450, family 3, subfamily A, polypeptide 5 | 6.63 | 2.71 |
| CYP3A5 | 205765_at | NM_000777 | cytochrome P450, family 3, subfamily A, polypeptide 5 | 5.99 | 2.57 |
| CYP3A5 | 214234_s_at | X90579 | cytochrome P450, family 3, subfamily A, polypeptide 5 | 5.99 | 2.56 |
| HLA-DMB | 203932_at | NM_002118 | major histocompatibility complex, class II, DM beta | 6.34 | 2.67 |
| CEACAM1 | 209498_at | X16354 | carcinoembryonic antigen-related cell adhesion molecule 1 (biliary glycoprotein) | 6.33 | 2.67 |
| C9orf152 | 229964_at | AI380443 | chromosome 9 open reading frame 152 | 6.28 | 2.66 |
| CCL20 | 205476_at | NM_004591 | chemokine (C-C motif) ligand 20 | 6.26 | 2.64 |
| FGB | 204988_at | NM_005141 | fibrinogen beta chain | 6.19 | 2.63 |
| FGB | 216238_s_at | BG545288 | fibrinogen beta chain | 5.94 | 2.56 |
| TFF1 | 205009_at | NM_003225 | trefoil factor 1 | 6.11 | 2.61 |
| KIAA1984 | 236518_at | BE208843 | KIAA1984 | 6.08 | 2.59 |
| C4BPA | 205654_at | NM_000715 | complement component 4 binding protein, alpha | 5.96 | 2.58 |
| TSPAN8 | 203824_at | NM_004616 | tetraspanin 8 | 5.96 | 2.56 |
| AHSG | 210929_s_at | AF130057 | alpha-2-HS-glycoprotein | 5.87 | 2.55 |
| TTLL6 | 230924_at | AI698574 | tubulin tyrosine ligase-like family, member 6 | 5.74 | 2.52 |
| ATF3 | 202672_s_at | NM_001674 | activating transcription factor 3 | 5.72 | 2.51 |
| CFB | 202357_s_at | NM_001710 | complement factor B | 5.51 | 2.46 |
| DUSP6 | 208891_at | BC003143 | dual specificity phosphatase 6 | 5.46 | 2.45 |
| DUSP6 | 208892_s_at | BC003143 | dual specificity phosphatase 6 | 5.13 | 2.36 |
| DUSP6 | 208893_s_at | BC005047 | dual specificity phosphatase 6 | 4.43 | 2.14 |
| LOC91461 | 225380_at | BF528878 | hypothetical protein BC007901 | 5.36 | 2.42 |
| KRT4 | 213240_s_at | X07695 | keratin 4 | 5.26 | 2.39 |
| ARRDC4 | 225283_at | AV701177 | arrestin domain containing 4 | 5.13 | 2.39 |
| SERPINA6 | 206325_at | NM_001756 | serpin peptidase inhibitor, clade A (alpha-1 antiproteinase, antitrypsin), member 6 | 5.10 | 2.34 |
| SEPP1 | 201427_s_at | NM_005410 | selenoprotein P, plasma, 1 | 5.08 | 2.34 |
| ANXA13 | 208323_s_at | NM_004306 | annexin A13 | 4.99 | 2.32 |
| APOH | 205216_s_at | NM_000042 | apolipoprotein H (beta-2-glycoprotein I) | 4.97 | 2.31 |
| KLF4 | 221841_s_at | BF514079 | Kruppel-like factor 4 (gut) | 4.93 | 2.29 |
| KLF4 | 220266_s_at | NM_004235 | Kruppel-like factor 4 (gut) | 3.46 | 1.79 |
| ST6GALNAC1 | 227725_at | Y11339 | ST6 (alpha-N-acetyl-neuraminyl-2,3-beta-galactosyl-1,3)-N-acetylgalactosaminide alpha-2,6-sialyltransferase 1 | 4.77 | 2.26 |
| TFPI | 210665_at | AF021834 | tissue factor pathway inhibitor (lipoprotein-associated coagulation inhibitor) | 4.76 | 2.25 |
| TFPI | 214378_at | BF109662 | tissue factor pathway inhibitor (lipoprotein-associated coagulation inhibitor) | 4.60 | 2.21 |
| TFPI | 209676_at | J03225 | tissue factor pathway inhibitor (lipoprotein-associated coagulation inhibitor) | 4.25 | 2.09 |
| TFPI | 210664_s_at | AF021834 | tissue factor pathway inhibitor (lipoprotein-associated coagulation inhibitor) | 3.24 | 1.70 |
| TFPI | 213258_at | BF511231 | tissue factor pathway inhibitor (lipoprotein-associated coagulation inhibitor) | 2.82 | 1.50 |
| NR4A2 | 216248_s_at | S77154 | nuclear receptor subfamily 4, group A, member 2 | 4.70 | 2.22 |
| NR4A2 | 204622_x_at | NM_006186 | nuclear receptor subfamily 4, group A, member 2 | 4.52 | 2.17 |
| NR4A2 | 204621_s_at | AI935096 | nuclear receptor subfamily 4, group A, member 2 | 4.18 | 2.06 |
| NR4A3 | 216979_at | X89894 | nuclear receptor subfamily 4, group A, member 3 | 4.64 | 2.21 |
| ERBB3 | 202454_s_at | NM_001982 | v-erb-b2 erythroblastic leukemia viral oncogene homolog 3 (avian) | 4.64 | 2.21 |
| ERBB3 | 226213_at | AV681807 | v-erb-b2 erythroblastic leukemia viral oncogene homolog 3 (avian) | 4.54 | 2.18 |
| ORM1/ORM2 | 205041_s_at | NM_000607 | orosomucoid 1///orosomucoid 2 | 4.62 | 2.20 |
| TMEM178 | 229302_at | AA058832 | transmembrane protein 178 | 4.62 | 2.19 |
| CDH17 | 209847_at | U07969 | cadherin 17, LI cadherin (liver-intestine) | 4.59 | 2.20 |
| FMO5 | 205776_at | NM_001461 | flavin containing monooxygenase 5 | 4.53 | 2.18 |
| MAFF | 36711_at | AL021977 | v-maf musculoaponeurotic fibrosarcoma oncogene homolog F (avian) | 4.49 | 2.16 |
| DDC | 205311_at | NM_000790 | dopa decarboxylase (aromatic L-amino acid decarboxylase) | 4.44 | 2.15 |
| LOC653879 | 217767_at | NM_000064 | similar to Complement C3 precursor | 4.43 | 2.15 |
| PLXNC1 | 213241_at | AF035307 | plexin C1 | 4.36 | 2.13 |
| RP5-1022P6.2 | 224835_at | AL109935 | hypothetical protein KIAA1434 | 4.29 | 2.10 |
| RP5-1022P6.2 | 224826_at | AK001947 | hypothetical protein KIAA1434 | 3.50 | 1.81 |
| RP5-1022P6.2 | 230492_s_at | BE328402 | hypothetical protein KIAA1434 | 2.98 | 1.58 |
| CORO2A | 227177_at | AL515381 | coronin, actin binding protein, 2A | 4.28 | 2.10 |
| CORO2A | 205538_at | NM_003389 | coronin, actin binding protein, 2A | 3.72 | 1.90 |
| TNFSF10 | 202688_at | NM_003810 | tumor necrosis factor (ligand) superfamily, member 10 | 4.27 | 2.10 |
| TNFSF10 | 202687_s_at | U57059 | tumor necrosis factor (ligand) superfamily, member 10 | 3.65 | 1.87 |
| TNFSF10 | 214329_x_at | AW474434 | tumor necrosis factor (ligand) superfamily, member 10 | 3.54 | 1.84 |
| FLJ32252 | 1557146_a_at | T03074 | hypothetical protein FLJ32252 | 4.27 | 2.09 |
| DKFZP564O0823 | 225809_at | AI659927 | DKFZP564O0823 protein | 4.26 | 2.09 |
| SLPI | 203021_at | NM_003064 | secretory leukocyte peptidase inhibitor | 4.26 | 2.09 |
| IFITM1 | 214022_s_at | AA749101 | interferon induced transmembrane protein 1 (9-27) | 4.19 | 2.06 |
| IFITM1 | 201601_x_at | NM_003641 | interferon induced transmembrane protein 1 (9-27) | 3.61 | 1.85 |
| MALAT1 | 224558_s_at | AI446756 | metastasis associated lung adenocarcinoma transcript 1 (non-coding RNA) | 4.15 | 2.04 |
| GBA3 | 222943_at | AW235567 | glucosidase, beta, acid 3 (cytosolic) | 4.12 | 2.04 |
| AQP3 | 39248_at | N74607 | aquaporin 3 (Gill blood group) | 4.12 | 2.04 |
| PLEKHH2 | 227148_at | AI913749 | pleckstrin homology domain containing, family H (with MyTH4 domain) member 2 | 4.07 | 2.02 |
| FGL1 | 205305_at | NM_004467 | fibrinogen-like 1 | 4.07 | 2.02 |
| CPB2 | 206651_s_at | NM_016413 | carboxypeptidase B2 (plasma) | 3.96 | 1.98 |
| HNMT | 204112_s_at | NM_006895 | histamine N-methyltransferase | 3.93 | 1.99 |
| NEB | 205054_at | NM_004543 | nebulin | 3.90 | 1.98 |
| ABCA8 | 204719_at | NM_007168 | ATP-binding cassette, sub-family A (ABC1), member 8 | 3.87 | 1.96 |
| ENTPD2 | 230430_at | AW134837 | ectonucleoside triphosphate diphosphohydrolase 2 | 3.87 | 1.94 |
| USH1C | 211184_s_at | AB006955 | Usher syndrome 1C (autosomal recessive, severe) | 3.86 | 1.95 |
| USH1C | 205137_x_at | NM_005709 | Usher syndrome 1C (autosomal recessive, severe) | 3.19 | 1.67 |
| HLA-DMA | 217478_s_at | X76775 | major histocompatibility complex, class II, DM alpha | 3.84 | 1.94 |
| ITIH2 | 204987_at | NM_002216 | inter-alpha (globulin) inhibitor H2 | 3.83 | 1.94 |
| PDZK1 | 205380_at | NM_002614 | PDZ domain containing 1 | 3.83 | 1.93 |
| CITED2 | 207980_s_at | NM_006079 | Cbp/p300-interacting transactivator, with Glu/Asp-rich carboxy-terminal domain, 2 | 3.83 | 1.94 |
| CITED2 | 209357_at | AF109161 | Cbp/p300-interacting transactivator, with Glu/Asp-rich carboxy-terminal domain, 2 | 3.55 | 1.83 |
| LOC648556 | 232696_at | AI394334 | uncharacterized gastric protein ZA43P | 3.80 | 1.93 |
| GSTA1 | 203924_at | NM_000846 | glutathione S-transferase A1 | 3.80 | 1.93 |
| ASGR1 | 206743_s_at | NM_001671 | asialoglycoprotein receptor 1 | 3.79 | 1.92 |
| PSCDBP | 209606_at | L06633 | pleckstrin homology, Sec7 and coiled-coil domains, binding protein | 3.68 | 1.87 |
| TMF1 | 242243_at | AI767435 | TATA element modulatory factor 1 | 3.67 | 1.87 |
| TOX3 | 214774_x_at | AK027006 | TOX high mobility group box family member 3 | 3.67 | 1.87 |
| TOX3 | 216623_x_at | AK025084 | TOX high mobility group box family member 3 | 3.55 | 1.82 |
| TOX3 | 215108_x_at | U80736 | TOX high mobility group box family member 3 | 3.35 | 1.74 |
| CFD | 205382_s_at | NM_001928 | complement factor D (adipsin) | 3.66 | 1.87 |
| SLC1A2 | 225491_at | AL157452 | solute carrier family 1 (glial high affinity glutamate transporter), member 2 | 3.65 | 1.84 |
| IGFBP1 | 205302_at | NM_000596 | insulin-like growth factor binding protein 1 | 3.63 | 1.85 |
| LOC145837 | 239594_at | BF110735 | hypothetical protein LOC145837 | 3.63 | 1.86 |
| PDK4 | 225207_at | AV707102 | pyruvate dehydrogenase kinase, isozyme 4 | 3.63 | 1.86 |
| PDK4 | 1562321_at | AL832708 | pyruvate dehydrogenase kinase, isozyme 4 | 2.62 | 1.39 |
| HAO1 | 220224_at | NM_017545 | hydroxyacid oxidase (glycolate oxidase) 1 | 3.62 | 1.86 |
| CNTNAP3 | 233202_at | AI433163 | contactin associated protein-like 3 | 3.62 | 1.86 |
| LOC692247 | 1569453_a_at | BG772667 | hypothetical locus LOC692247 | 3.53 | 1.82 |
| ID2/ID2B | 213931_at | AI819238 | inhibitor of DNA binding 2, dominant negative helix-loop-helix protein///inhibitor of DNA binding 2B, dominant negative helix-loop-helix protein | 3.53 | 1.80 |
| SLC16A6 | 230748_at | AI873273 | solute carrier family 16, member 6 (monocarboxylic acid transporter 7) | 3.51 | 1.83 |
| PLA1A | 219584_at | NM_015900 | phospholipase A1 member A | 3.50 | 1.81 |
| CFH | 213800_at | X04697 | complement factor H | 3.48 | 1.80 |
| C6orf86 | 242055_at | AW136397 | Chromosome 6 open reading frame 86 | 3.47 | 1.80 |
| EHF | 225645_at | AI763378 | Ets homologous factor | 3.44 | 1.79 |
| SLC4A4 | 203908_at | NM_003759 | solute carrier family 4, sodium bicarbonate cotransporter, member 4 | 3.43 | 1.76 |
| TM4SF4 | 209937_at | BC001386 | transmembrane 4 L six family member 4 | 3.41 | 1.77 |
| C12orf39 | 229778_at | BE326710 | chromosome 12 open reading frame 39 | 3.38 | 1.77 |
| KCNJ2 | 206765_at | AF153820 | potassium inwardly-rectifying channel, subfamily J, member 2 | 3.37 | 1.77 |
| WNT4 | 208606_s_at | NM_030761 | wingless-type MMTV integration site family, member 4 | 3.37 | 1.75 |
| SLC40A1 | 223044_at | AL136944 | solute carrier family 40 (iron-regulated transporter), member 1 | 3.36 | 1.75 |
| S100P | 204351_at | NM_005980 | S100 calcium binding protein P | 3.33 | 1.74 |
| MED31 | 236241_at | BF593977 | mediator complex subunit 31 | 3.33 | 1.73 |
| SEMA4G | 219194_at | NM_017893 | sema domain, immunoglobulin domain (Ig), transmembrane domain (TM) and short cytoplasmic domain, (semaphorin) 4G | 3.33 | 1.73 |
| VIL1 | 228912_at | AI436136 | Villin 1 | 3.33 | 1.73 |
| PDE4B | 215671_at | AU144792 | phosphodiesterase 4B, cAMP-specific (phosphodiesterase E4 dunce homolog, Drosophila) | 3.29 | 1.72 |
| LGALS4 | 204272_at | NM_006149 | lectin, galactoside-binding, soluble, 4 (galectin 4) | 3.28 | 1.72 |
| PTGS2 | 1554997_a_at | AY151286 | prostaglandin-endoperoxide synthase 2 (prostaglandin G/H synthase and cyclooxygenase) | 3.25 | 1.70 |
| PTGS2 | 204748_at | NM_000963 | prostaglandin-endoperoxide synthase 2 (prostaglandin G/H synthase and cyclooxygenase) | 3.04 | 1.61 |
| IL8 | 202859_x_at | NM_000584 | interleukin 8 | 3.22 | 1.68 |
| HK2 | 202934_at | AI761561 | hexokinase 2 | 3.17 | 1.67 |
| FOS | 209189_at | BC004490 | v-fos FBJ murine osteosarcoma viral oncogene homolog | 3.17 | 1.67 |
| TM4SF5 | 206242_at | NM_003963 | transmembrane 4 L six family member 5 | 3.16 | 1.66 |
| FLJ37512 | 244065_at | AW016751 | similar to Contactin-associated protein-like 3 precursor (Cell recognition molecule Caspr3) | 3.16 | 1.66 |
| SLC23A1 | 223732_at | AF170911 | solute carrier family 23 (nucleobase transporters), member 1 | 3.15 | 1.65 |
| SELENBP1 | 214433_s_at | NM_003944 | selenium binding protein 1 | 3.07 | 1.62 |
| HES1 | 203394_s_at | BE973687 | hairy and enhancer of split 1, (Drosophila) | 3.07 | 1.61 |
| PCSK6 | 242662_at | AI056815 | Proprotein convertase subtilisin/kexin type 6 | 3.06 | 1.61 |
| PCSK6 | 207414_s_at | NM_002570 | proprotein convertase subtilisin/kexin type 6 | 2.82 | 1.50 |
| CLDN2 | 223509_at | AF177340 | claudin 2 | 3.06 | 1.60 |
| FGFR3 | 204379_s_at | NM_000142 | fibroblast growth factor receptor 3 (achondroplasia, thanatophoric dwarfism) | 3.05 | 1.60 |
| C5 | 205500_at | NM_001735 | complement component 5 | 3.04 | 1.60 |
| MYLIP | 228097_at | AW292746 | myosin regulatory light chain interacting protein | 3.04 | 1.60 |
| FN1 | 212464_s_at | X02761 | fibronectin 1 | 3.02 | 1.59 |
| FN1 | 216442_x_at | AK026737 | fibronectin 1 | 2.99 | 1.58 |
| FN1 | 210495_x_at | AF130095 | fibronectin 1 | 2.96 | 1.56 |
| FN1 | 211719_x_at | BC005858 | fibronectin 1 | 2.95 | 1.56 |
| CYP4F3 | 206515_at | NM_000896 | cytochrome P450, family 4, subfamily F, polypeptide 3 | 3.01 | 1.59 |
| CPN1 | 206256_at | NM_001308 | carboxypeptidase N, polypeptide 1 | 3.01 | 1.59 |
| SPRY1 | 212558_at | BF508662 | sprouty homolog 1, antagonist of FGF signaling (Drosophila) | 2.99 | 1.58 |
| RHOBTB1 | 212651_at | AB018283 | Rho-related BTB domain containing 1 | 2.98 | 1.57 |
| CA9 | 205199_at | NM_001216 | carbonic anhydrase IX | 2.95 | 1.57 |
| TSC22D3 | 208763_s_at | AL110191 | TSC22 domain family, member 3 | 2.95 | 1.56 |
| PLXNA2 | 213030_s_at | AI688418 | plexin A2 | 2.93 | 1.56 |
| IFITM2 | 201315_x_at | NM_006435 | interferon induced transmembrane protein 2 (1-8D) | 2.91 | 1.53 |
| XBP1 | 200670_at | NM_005080 | X-box binding protein 1 | 2.90 | 1.54 |
| TM4SF20 | 220639_at | NM_024795 | transmembrane 4 L six family member 20 | 2.90 | 1.54 |
| TMED10 | 238886_at | BF056141 | Transmembrane emp24-like trafficking protein 10 (yeast) | 2.90 | 1.53 |
| CEACAM6 | 203757_s_at | BC005008 | carcinoembryonic antigen-related cell adhesion molecule 6 (non-specific cross reacting antigen) | 2.88 | 1.52 |
| CEACAM6 | 211657_at | M18728 | carcinoembryonic antigen-related cell adhesion molecule 6 (non-specific cross reacting antigen) | 2.63 | 1.40 |
| RARRES1 | 221872_at | AI669229 | retinoic acid receptor responder (tazarotene induced) 1 | 2.87 | 1.52 |
| RARRES1 | 206392_s_at | NM_002888 | retinoic acid receptor responder (tazarotene induced) 1 | 2.63 | 1.39 |
| BACH1 | 204194_at | NM_001186 | BTB and CNC homology 1, basic leucine zipper transcription factor 1 | 2.84 | 1.51 |
| NR4A1 | 202340_x_at | NM_002135 | nuclear receptor subfamily 4, group A, member 1 | 2.83 | 1.50 |
| ANXA1 | 233011_at | AU155094 | Annexin A1 | 2.82 | 1.49 |
| C4orf18 | 223204_at | AF260333 | chromosome 4 open reading frame 18 | 2.81 | 1.49 |
| NOSTRIN | 226992_at | AK002203 | nitric oxide synthase trafficker | 2.78 | 1.47 |
| OSTbeta | 230830_at | AI479168 | organic solute transporter beta | 2.77 | 1.47 |
| CAPN5 | 226292_at | BF195709 | calpain 5 | 2.71 | 1.44 |
| QPRT | 204044_at | NM_014298 | quinolinate phosphoribosyltransferase (nicotinate-nucleotide pyrophosphorylase (carboxylating)) | 2.70 | 1.43 |
| SLC44A5 | 235763_at | AA001450 | solute carrier family 44, member 5 | 2.70 | 1.43 |
| LCN2 | 212531_at | NM_005564 | lipocalin 2 (oncogene 24p3) | 2.67 | 1.42 |
| RPL31 | 200962_at | AI348010 | ribosomal protein L31 | 2.67 | 1.42 |
| PPP1R15A | 202014_at | NM_014330 | protein phosphatase 1, regulatory (inhibitor) subunit 15A | 2.67 | 1.41 |
| NFKBIZ | 223218_s_at | AB037925 | nuclear factor of kappa light polypeptide gene enhancer in B-cells inhibitor, zeta | 2.66 | 1.42 |
| ANK3 | 206385_s_at | NM_020987 | ankyrin 3, node of Ranvier (ankyrin G) | 2.66 | 1.41 |
| LOC284454 | 1555847_a_at | BU617052 | hypothetical protein LOC284454 | 2.62 | 1.39 |
| KLF6 | 1555832_s_at | BU683415 | Kruppel-like factor 6 | 2.62 | 1.39 |
| F7 | 207300_s_at | NM_000131 | coagulation factor VII (serum prothrombin conversion accelerator) | 2.61 | 1.39 |
| MAOA | 212741_at | AA923354 | monoamine oxidase A | 2.60 | 1.38 |
| CD302 | 203799_at | NM_014880 | CD302 molecule | 2.60 | 1.38 |
| RNASE4 | 205158_at | NM_002937 | ribonuclease, RNase A family, 4 | 2.51 | 1.33 |
| LOC202134/LOC653316/NY-REN-7 | 214945_at | AW514267 | NY-REN-7 antigen///hypothetical protein LOC202134///hypothetical protein LOC653316 | 2.50 | 1.32 |
| PROS1 | 207808_s_at | NM_000313 | protein S (alpha) | 2.46 | 1.30 |
| EEF1A1 | 227708_at | AW469790 | eukaryotic translation elongation factor 1 alpha 1 | 2.45 | 1.29 |
| TGFBR2 | 208944_at | D50683 | transforming growth factor, beta receptor II (70/80kDa) | 2.43 | 1.28 |
| BCL6 | 203140_at | NM_001706 | B-cell CLL/lymphoma 6 (zinc finger protein 51) | 2.36 | 1.24 |
| MAP3K1 | 225927_at | AA541479 | mitogen-activated protein kinase kinase kinase 1 | 2.35 | 1.23 |
| RNASE4 | 213397_x_at | AI761728 | ribonuclease, RNase A family, 4 | 2.34 | 1.23 |
| C5orf26 | 225698_at | BF314746 | chromosome 5 open reading frame 26 | 2.34 | 1.23 |
| GPR37 | 214586_at | T16257 | G protein-coupled receptor 37 (endothelin receptor type B-like) | 2.33 | 1.22 |
| SLC23A2 | 209236_at | AL389886 | solute carrier family 23 (nucleobase transporters), member 2 | 2.30 | 1.20 |
| ST6GAL1 | 201998_at | AI743792 | ST6 beta-galactosamide alpha-2,6-sialyltranferase 1 | 2.27 | 1.18 |
| ANG | 205141_at | NM_001145 | angiogenin, ribonuclease, RNase A family, 5 | 2.25 | 1.17 |
|  |  |  |  |  |  |
| **Genes downregulated in 3D versus 2D** | | |  |  |  |
| BCL2A1 | 205681_at | NM_004049 | BCL2-related protein A1 | -13.20 | -3.76 |
| GREM1 | 218468_s_at | AF154054 | gremlin 1, cysteine knot superfamily, homolog (Xenopus laevis) | -12.68 | -3.66 |
| GREM1 | 218469_at | NM_013372 | gremlin 1, cysteine knot superfamily, homolog (Xenopus laevis) | -11.43 | -3.52 |
| SPOCK1 | 202363_at | AF231124 | sparc/osteonectin, cwcv and kazal-like domains proteoglycan (testican) 1 | -12.43 | -3.64 |
| TMEPAI | 222450_at | AL035541 | transmembrane, prostate androgen induced RNA | -10.55 | -3.41 |
| TMEPAI | 222449_at | AL035541 | transmembrane, prostate androgen induced RNA | -9.93 | -3.32 |
| TMEPAI | 217875_s_at | NM_020182 | transmembrane, prostate androgen induced RNA | -7.50 | -2.91 |
| HAS2 | 206432_at | NM_005328 | hyaluronan synthase 2 | -10.10 | -3.34 |
| MYOCD | 237206_at | AI452798 | myocardin | -10.05 | -3.33 |
| LBH | 221011_s_at | NM_030915 | limb bud and heart development homolog (mouse) | -8.88 | -3.15 |
| COL5A1 | 203325_s_at | AI130969 | collagen, type V, alpha 1 | -7.92 | -2.98 |
| COL5A1 | 212488_at | N30339 | collagen, type V, alpha 1 | -6.15 | -2.62 |
| COL5A1 | 212489_at | AI983428 | collagen, type V, alpha 1 | -5.28 | -2.40 |
| CDH4 | 220227_at | NM_024883 | cadherin 4, type 1, R-cadherin (retinal) | -7.79 | -2.98 |
| NPTX1 | 204684_at | NM_002522 | neuronal pentraxin I | -7.69 | -2.95 |
| PLAU | 211668_s_at | K03226 | plasminogen activator, urokinase | -7.35 | -2.94 |
| PLAU | 205479_s_at | NM_002658 | plasminogen activator, urokinase | -5.23 | -2.39 |
| GLIPR1 | 226136_at | N32834 | GLI pathogenesis-related 1 (glioma) | -7.10 | -2.84 |
| GLIPR1 | 226142_at | AV682252 | GLI pathogenesis-related 1 (glioma) | -6.28 | -2.65 |
| GLIPR1 | 204222_s_at | NM_006851 | GLI pathogenesis-related 1 (glioma) | -5.81 | -2.55 |
| TNFAIP6 | 206026_s_at | NM_007115 | tumor necrosis factor, alpha-induced protein 6 | -6.87 | -2.78 |
| TNFAIP6 | 206025_s_at | AW188198 | tumor necrosis factor, alpha-induced protein 6 | -5.28 | -2.33 |
| DIRAS3 | 215506_s_at | AK021882 | DIRAS family, GTP-binding RAS-like 3 | -6.84 | -2.77 |
| ITGB6 | 226535_at | AK026736 | integrin, beta 6 | -6.78 | -2.77 |
| IL11 | 206924_at | NM_000641 | interleukin 11 | -6.77 | -2.77 |
| DIO2 | 203700_s_at | NM_013989 | deiodinase, iodothyronine, type II | -6.41 | -2.74 |
| DIO2 | 203699_s_at | U53506 | deiodinase, iodothyronine, type II | -5.19 | -2.37 |
| NPR3 | 219790_s_at | NM_000908 | natriuretic peptide receptor C/guanylate cyclase C (atrionatriuretic peptide receptor C) | -5.70 | -2.49 |
| NPR3 | 219789_at | AI628360 | natriuretic peptide receptor C/guanylate cyclase C (atrionatriuretic peptide receptor C) | -4.30 | -2.13 |
| BCAT1 | 214452_at | NM_005504 | branched chain aminotransferase 1, cytosolic | -5.29 | -2.40 |
| BCAT1 | 225285_at | AK025615 | branched chain aminotransferase 1, cytosolic | -3.18 | -1.67 |
| BCAT1 | 226517_at | AL390172 | branched chain aminotransferase 1, cytosolic | -3.07 | -1.63 |
| MOBKL2B | 229568_at | AI692878 | MOB1, Mps One Binder kinase activator-like 2B (yeast) | -5.27 | -2.40 |
| MOBKL2B | 226844_at | AI375115 | MOB1, Mps One Binder kinase activator-like 2B (yeast) | -3.64 | -1.87 |
| CXCR7 | 212977_at | AI817041 | chemokine (C-X-C motif) receptor 7 | -5.27 | -2.41 |
| C15orf48 | 223484_at | AF228422 | chromosome 15 open reading frame 48 | -5.19 | -2.40 |
| EDG3 | 228176_at | AA534817 | endothelial differentiation, sphingolipid G-protein-coupled receptor, 3 | -5.04 | -2.35 |
| AFAP1L2 | 226829_at | AW138743 | actin filament associated protein 1-like 2 | -4.94 | -2.33 |
| RNF182 | 230720_at | AI884906 | ring finger protein 182 | -4.90 | -2.32 |
| IGFBP7 | 201162_at | NM_001553 | insulin-like growth factor binding protein 7 | -4.73 | -2.24 |
| IGFBP7 | 201163_s_at | NM_001553 | insulin-like growth factor binding protein 7 | -4.72 | -2.24 |
| KRT80 | 231849_at | AL162069 | keratin 80 | -4.52 | -2.19 |
| ANXA10 | 210143_at | AF196478 | annexin A10 | -4.44 | -2.20 |
| TNFRSF12A | 218368_s_at | NM_016639 | tumor necrosis factor receptor superfamily, member 12A | -4.43 | -2.18 |
| RDX | 204969_s_at | NM_002906 | radixin | -4.42 | -2.15 |
| RDX | 212398_at | AI057093 | radixin | -3.36 | -1.75 |
| KCNMA1 | 221583_s_at | AI129381 | potassium large conductance calcium-activated channel, subfamily M, alpha member 1 | -4.41 | -2.14 |
| KCNMA1 | 221584_s_at | U11058 | potassium large conductance calcium-activated channel, subfamily M, alpha member 1 | -2.79 | -1.48 |
| CCDC80 | 225242_s_at | AW303375 | coiled-coil domain containing 80 | -4.40 | -2.15 |
| PAQR5 | 220333_at | NM_017705 | progestin and adipoQ receptor family member V | -4.37 | -2.14 |
| SPARC | 200665_s_at | NM_003118 | secreted protein, acidic, cysteine-rich (osteonectin) | -4.27 | -2.09 |
| GNG2 | 224964_s_at | AK026424 | guanine nucleotide binding protein (G protein), gamma 2 | -4.24 | -2.11 |
| FBN2 | 203184_at | NM_001999 | fibrillin 2 (congenital contractural arachnodactyly) | -4.22 | -2.08 |
| IGFBP6 | 203851_at | NM_002178 | insulin-like growth factor binding protein 6 | -4.08 | -2.03 |
| FLRT2 | 204359_at | NM_013231 | fibronectin leucine rich transmembrane protein 2 | -4.04 | -2.02 |
| OSAP | 223734_at | AF329088 | ovary-specific acidic protein | -4.02 | -2.02 |
| LYPD1 | 212909_at | AL567376 | LY6/PLAUR domain containing 1 | -3.98 | -2.01 |
| P4HA3 | 228703_at | AW665086 | procollagen-proline, 2-oxoglutarate 4-dioxygenase (proline 4-hydroxylase), alpha polypeptide III | -3.96 | -1.99 |
| SOCS2 | 203372_s_at | AB004903 | suppressor of cytokine signaling 2 | -3.95 | -1.98 |
| SLC7A11 | 207528_s_at | NM_014331 | solute carrier family 7, (cationic amino acid transporter, y+ system) member 11 | -3.79 | -1.93 |
| PLCE1 | 205111_s_at | NM_016341 | phospholipase C, epsilon 1 | -3.77 | -1.91 |
| PGM2L1 | 229553_at | AA736452 | phosphoglucomutase 2-like 1 | -3.74 | -1.91 |
| NAV3 | 204823_at | NM_014903 | neuron navigator 3 | -3.73 | -1.90 |
| NAV3 | 1552658_a_at | NM_014903 | neuron navigator 3 | -3.34 | -1.73 |
| DST | 204455_at | NM_001723 | dystonin | -3.71 | -1.89 |
| MAF | 209348_s_at | AF055376 | v-maf musculoaponeurotic fibrosarcoma oncogene homolog (avian) | -3.70 | -1.89 |
| SERPINE1 | 202628_s_at | NM_000602 | serpin peptidase inhibitor, clade E (nexin, plasminogen activator inhibitor type 1), member 1 | -3.68 | -1.89 |
| LOXL2 | 202998_s_at | NM_002318 | lysyl oxidase-like 2 | -3.62 | -1.86 |
| SCD | 211708_s_at | BC005807 | stearoyl-CoA desaturase (delta-9-desaturase) | -3.59 | -1.86 |
| PLAT | 201860_s_at | NM_000930 | plasminogen activator, tissue | -3.57 | -1.84 |
| ADRB1 | 229309_at | AI625747 | adrenergic, beta-1-, receptor | -3.54 | -1.83 |
| HERC5 | 219863_at | NM_016323 | hect domain and RLD 5 | -3.51 | -1.81 |
| SEPT6 | 1555526_a_at | AF403061 | septin 6 | -3.51 | -1.81 |
| SEPT6 | 212415_at | AW150913 | septin 6 | -2.79 | -1.48 |
| PDXK | 202671_s_at | NM_003681 | pyridoxal (pyridoxine, vitamin B6) kinase | -3.46 | -1.80 |
| NEXN | 1552309_a_at | NM_144573 | nexilin (F actin binding protein) | -3.45 | -1.79 |
| NEXN | 226103_at | AF114264 | nexilin (F actin binding protein) | -3.30 | -1.72 |
| NUPR1 | 209230_s_at | AF135266 | nuclear protein 1 | -3.42 | -1.78 |
| FHL1 | 214505_s_at | AF220153 | four and a half LIM domains 1 | -3.41 | -1.78 |
| FHL1 | 210299_s_at | AF063002 | four and a half LIM domains 1 | -3.37 | -1.76 |
| FHL1 | 201540_at | NM_001449 | four and a half LIM domains 1 | -2.84 | -1.51 |
| TIMP2 | 224560_at | BF107565 | TIMP metallopeptidase inhibitor 2 | -3.39 | -1.76 |
| TIMP2 | 203167_at | NM_003255 | TIMP metallopeptidase inhibitor 2 | -2.92 | -1.55 |
| TIMP2 | 231579_s_at | BE968786 | TIMP metallopeptidase inhibitor 2 | -2.87 | -1.52 |
| WDR1 | 210935_s_at | AF274954 | WD repeat domain 1 | -3.38 | -1.77 |
| APBB1IP | 230925_at | AI093231 | amyloid beta (A4) precursor protein-binding, family B, member 1 interacting protein | -3.38 | -1.76 |
| AKAP2/PALM2-AKAP2 | 226694_at | BG540494 | A kinase (PRKA) anchor protein 2///PALM2-AKAP2 protein | -3.37 | -1.75 |
| ANKRD29 | 238332_at | AI307802 | ankyrin repeat domain 29 | -3.36 | -1.76 |
| ITGA4 | 213416_at | BG532690 | integrin, alpha 4 (antigen CD49D, alpha 4 subunit of VLA-4 receptor) | -3.25 | -1.71 |
| COL4A1 | 211981_at | NM_001845 | collagen, type IV, alpha 1 | -3.21 | -1.68 |
| SAMD9 | 228531_at | AA741307 | sterile alpha motif domain containing 9 | -3.20 | -1.68 |
| NRP2 | 214632_at | AA295257 | neuropilin 2 | -3.19 | -1.68 |
| NRP2 | 211844_s_at | AF022859 | neuropilin 2 | -3.06 | -1.62 |
| KIF3C | 203390_s_at | NM_002254 | kinesin family member 3C | -3.15 | -1.65 |
| SCG2 | 204035_at | NM_003469 | secretogranin II (chromogranin C) | -3.13 | -1.65 |
| SKIL | 206675_s_at | NM_005414 | SKI-like oncogene | -3.12 | -1.64 |
| HMOX1 | 203665_at | NM_002133 | heme oxygenase (decycling) 1 | -3.12 | -1.64 |
| PRSS23 | 229441_at | AI569872 | Protease, serine, 23 | -3.10 | -1.64 |
| ADAM19 | 209765_at | Y13786 | ADAM metallopeptidase domain 19 (meltrin beta) | -3.02 | -1.60 |
| BNC2 | 220272_at | NM_017637 | basonuclin 2 | -3.00 | -1.59 |
| CADM1 | 209030_s_at | NM_014333 | cell adhesion molecule 1 | -2.94 | -1.55 |
| CADM1 | 209031_at | AL519710 | cell adhesion molecule 1 | -2.59 | -1.37 |
| PADI2 | 209791_at | AL049569 | peptidyl arginine deiminase, type II | -2.92 | -1.54 |
| EFHB | 239477_at | BF941046 | EF-hand domain family, member B | -2.91 | -1.54 |
| DSE | 218854_at | NM_013352 | dermatan sulfate epimerase | -2.83 | -1.50 |
| LHFP | 218656_s_at | NM_005780 | lipoma HMGIC fusion partner | -2.82 | -1.50 |
| CDC42EP3 | 209286_at | AI754416 | CDC42 effector protein (Rho GTPase binding) 3 | -2.77 | -1.47 |
| CDC42EP3 | 209288_s_at | AL136842 | CDC42 effector protein (Rho GTPase binding) 3 | -2.67 | -1.42 |
| LOC401097 | 236738_at | AW057589 | Similar to LOC166075 | -2.74 | -1.46 |
| TPM1 | 206116_s_at | NM_000366 | tropomyosin 1 (alpha) | -2.74 | -1.46 |
| EPHB2 | 211165_x_at | D31661 | EPH receptor B2 | -2.72 | -1.45 |
| C1orf43 | 1555225_at | BC008306 | chromosome 1 open reading frame 43 | -2.66 | -1.41 |
| MRPS12 | 204330_s_at | AA587905 | mitochondrial ribosomal protein S12 | -2.64 | -1.40 |
| NPC1 | 202679_at | NM_000271 | Niemann-Pick disease, type C1 | -2.62 | -1.39 |
| MFAP3L | 205442_at | NM_021647 | microfibrillar-associated protein 3-like | -2.62 | -1.39 |
| N-PAC/SEPT6 | 212414_s_at | D50918 | cytokine-like nuclear factor n-pac///septin 6 | -2.57 | -1.36 |
| THBD | 203888_at | NM_000361 | thrombomodulin | -2.57 | -1.36 |
| RSPO3 | 228186_s_at | BF589322 | R-spondin 3 homolog (Xenopus laevis) | -2.54 | -1.35 |
| PLEK2 | 218644_at | NM_016445 | pleckstrin 2 | -2.47 | -1.30 |
| NAV2 | 218330_s_at | NM_018162 | neuron navigator 2 | -2.46 | -1.30 |
| C8orf13 | 226614_s_at | BE856336 | chromosome 8 open reading frame 13 | -2.45 | -1.29 |
| TGFBI | 201506_at | NM_000358 | transforming growth factor, beta-induced, 68kDa | -2.43 | -1.28 |
| COTL1 | 221059_s_at | NM_021615 | coactosin-like 1 (Dictyostelium) | -2.35 | -1.23 |

^a^Listing of up- and downregulated genes according to SAM.
