# Supplementary material for: Genome-Wide Gene Expression Analysis in Cancer Cells Reveals 3D Growth to Affect ECM and Processes Associated with Cell Adhesion but Not DNA Repair
Source: PLoS One. 2012 Apr 11;7(4):e34279. doi: 10.1371/journal.pone.0034279 (PMC3324525; doi:10.1371/journal.pone.0034279)
Supplement: Table S2 — Gene expression analysis in 3D versus 2D UT-SCC15 cell cultures. (DOC) [file pone.0034279.s002.doc]

| **Table S2. Gene expression analysis in 3D versus 2D UT-SCC15 cell cultures.^a^** | | | |  |  |
| --- | --- | --- | --- | --- | --- |
|  |  |  |  |  |  |
| Common name | Affymetrix-ID | Genbank | Description | Fold change | Signal log ratio |
| **Genes upregulated in 3D versus 2D** | | |  |  |  |
| FABP4 | 203980_at | NM_001442 | fatty acid binding protein 4, adipocyte | 8.42 | 3.08 |
| TXNIP | 201010_s_at | NM_006472 | thioredoxin interacting protein | 8.04 | 3.01 |
| TXNIP | 201009_s_at | AI439556 | thioredoxin interacting protein | 5.38 | 2.43 |
| TXNIP | 201008_s_at | AA812232 | thioredoxin interacting protein | 4.36 | 2.13 |
| MAF | 209348_s_at | AF055376 | v-maf musculoaponeurotic fibrosarcoma oncogene homolog (avian) | 7.44 | 2.89 |
| MAF | 206363_at | NM_005360 | v-maf musculoaponeurotic fibrosarcoma oncogene homolog (avian) | 5.54 | 2.47 |
| IFI6 | 204415_at | NM_022873 | interferon, alpha-inducible protein 6 | 5.97 | 2.59 |
| INHBA | 210511_s_at | M13436 | inhibin, beta A | 5.93 | 2.56 |
| ARRDC3 | 224797_at | AB037797 | arrestin domain containing 3 | 5.92 | 2.58 |
| MMP28 | 219909_at | NM_024302 | matrix metallopeptidase 28 | 5.73 | 2.46 |
| MMP28 | 239272_at | AI927208 | matrix metallopeptidase 28 | 4.00 | 2.00 |
| MMP28 | 239273_s_at | AI927208 | matrix metallopeptidase 28 | 3.66 | 1.90 |
| FEZ1 | 203562_at | NM_005103 | fasciculation and elongation protein zeta 1 (zygin I) | 5.51 | 2.46 |
| EGLN3 | 219232_s_at | NM_022073 | egl nine homolog 3 (C. elegans) | 5.13 | 2.36 |
| EGLN3 | 222847_s_at | AI378406 | egl nine homolog 3 (C. elegans) | 3.14 | 1.65 |
| IFI44L | 204439_at | NM_006820 | interferon-induced protein 44-like | 4.94 | 2.32 |
| CCL5 | 1555759_a_at | AF043341 | chemokine (C-C motif) ligand 5 | 4.83 | 2.29 |
| CCL5 | 1405_i_at | M21121 | chemokine (C-C motif) ligand 5 | 4.76 | 2.25 |
| CCL5 | 204655_at | NM_002985 | chemokine (C-C motif) ligand 5 | 4.64 | 2.21 |
| BST2 | 201641_at | NM_004335 | bone marrow stromal cell antigen 2 | 4.63 | 2.21 |
| CP | 204846_at | NM_000096 | ceruloplasmin (ferroxidase) | 4.51 | 2.16 |
| CP | 227253_at | AI922198 | ceruloplasmin (ferroxidase) | 3.15 | 1.64 |
| WFDC5 | 242204_at | AI242082 | WAP four-disulfide core domain 5 | 4.37 | 2.14 |
| ARRDC4 | 225283_at | AV701177 | arrestin domain containing 4 | 4.29 | 2.11 |
| TMEPAI | 222449_at | AL035541 | transmembrane, prostate androgen induced RNA | 4.24 | 2.07 |
| TMEPAI | 222450_at | AL035541 | transmembrane, prostate androgen induced RNA | 3.33 | 1.73 |
| DNAH17 | 214229_at | AL122077 | dynein, axonemal, heavy chain 17 | 4.23 | 2.10 |
| PRDM1 | 228964_at | AI692659 | PR domain containing 1, with ZNF domain | 4.16 | 2.06 |
| NEDD9 | 202149_at | AL136139 | neural precursor cell expressed, developmentally down-regulated 9 | 4.16 | 2.03 |
| MX2 | 204994_at | NM_002463 | myxovirus (influenza virus) resistance 2 (mouse) | 4.12 | 2.04 |
| SERPINA3 | 202376_at | NM_001085 | serpin peptidase inhibitor, clade A (alpha-1 antiproteinase, antitrypsin), member 3 | 4.12 | 2.04 |
| ANKRD38 | 229125_at | AA456955 | ankyrin repeat domain 38 | 4.01 | 1.98 |
| SCN4B | 236359_at | AW026241 | sodium channel, voltage-gated, type IV, beta | 3.97 | 1.98 |
| PAPPA | 228128_x_at | AI110886 | pregnancy-associated plasma protein A, pappalysin 1 | 3.93 | 1.98 |
| PAPPA | 224941_at | BF107618 | pregnancy-associated plasma protein A, pappalysin 1 | 3.25 | 1.72 |
| CXCL11 | 211122_s_at | AF002985 | chemokine (C-X-C motif) ligand 11 | 3.78 | 1.91 |
| SLC28A3 | 232277_at | AA643687 | solute carrier family 28 (sodium-coupled nucleoside transporter), member 3 | 3.76 | 1.92 |
| TGM2 | 201042_at | AL031651 | transglutaminase 2 (C polypeptide, protein-glutamine-gamma-glutamyltransferase) | 3.69 | 1.87 |
| RPS6KA2 | 212912_at | AI992251 | ribosomal protein S6 kinase, 90kDa, polypeptide 2 | 3.56 | 1.84 |
| ASB2 | 227915_at | AI872284 | ankyrin repeat and SOCS box-containing 2 | 3.56 | 1.83 |
| LTBP2 | 223690_at | AF113211 | latent transforming growth factor beta binding protein 2 | 3.51 | 1.81 |
| LTBP2 | 204682_at | NM_000428 | latent transforming growth factor beta binding protein 2 | 2.40 | 1.26 |
| GBP5 | 238581_at | BG271923 | guanylate binding protein 5 | 3.47 | 1.80 |
| CA12 | 203963_at | NM_001218 | carbonic anhydrase XII | 3.46 | 1.79 |
| CA12 | 204508_s_at | BC001012 | carbonic anhydrase XII | 3.35 | 1.74 |
| CA12 | 215867_x_at | AL050025 | carbonic anhydrase XII | 2.53 | 1.34 |
| CA12 | 214164_x_at | BF752277 | carbonic anhydrase XII | 2.51 | 1.33 |
| LAMC2 | 202267_at | NM_005562 | laminin, gamma 2 | 3.41 | 1.76 |
| TIMP3 | 201150_s_at | NM_000362 | TIMP metallopeptidase inhibitor 3 (Sorsby fundus dystrophy, pseudoinflammatory) | 3.38 | 1.75 |
| OAS2 | 204972_at | NM_016817 | 2'-5'-oligoadenylate synthetase 2, 69/71kDa | 3.34 | 1.75 |
| PTHLH | 211756_at | BC005961 | parathyroid hormone-like hormone | 3.33 | 1.74 |
| PTHLH | 206300_s_at | NM_002820 | parathyroid hormone-like hormone | 3.24 | 1.70 |
| PTHLH | 210355_at | J03580 | parathyroid hormone-like hormone | 3.09 | 1.63 |
| XAF1 | 242234_at | AI859280 | XIAP associated factor-1 | 3.28 | 1.72 |
| XAF1 | 228617_at | AA142842 | XIAP associated factor-1 | 2.46 | 1.30 |
| ZBTB20 | 205383_s_at | NM_015642 | zinc finger and BTB domain containing 20 | 3.27 | 1.72 |
| KIAA1644 | 52837_at | AL047020 | KIAA1644 protein | 3.17 | 1.66 |
| SERPINE1 | 202628_s_at | NM_000602 | serpin peptidase inhibitor, clade E (nexin, plasminogen activator inhibitor type 1), member 1 | 3.16 | 1.65 |
| SERPINE1 | 202627_s_at | AL574210 | serpin peptidase inhibitor, clade E (nexin, plasminogen activator inhibitor type 1), member 1 | 2.91 | 1.53 |
| LAMA3 | 203726_s_at | NM_000227 | laminin, alpha 3 | 3.15 | 1.65 |
| KCTD11 | 235857_at | AI859242 | potassium channel tetramerisation domain containing 11 | 3.14 | 1.65 |
| COL12A1 | 225664_at | AA788946 | collagen, type XII, alpha 1 | 3.14 | 1.65 |
| COL12A1 | 231766_s_at | U73778 | collagen, type XII, alpha 1 | 3.05 | 1.61 |
| VLDLR | 209822_s_at | L22431 | very low density lipoprotein receptor | 3.03 | 1.60 |
| VNN1 | 205844_at | NM_004666 | vanin 1 | 3.03 | 1.60 |
| TMPRSS3 | 223949_at | AB038160 | transmembrane protease, serine 3 | 3.02 | 1.60 |
| NDRG1 | 200632_s_at | NM_006096 | N-myc downstream regulated gene 1 | 2.95 | 1.56 |
| RRAS | 212647_at | NM_006270 | related RAS viral (r-ras) oncogene homolog | 2.95 | 1.56 |
| LOC129607 | 226702_at | AI742057 | hypothetical protein LOC129607 | 2.93 | 1.55 |
| MX1 | 202086_at | NM_002462 | myxovirus (influenza virus) resistance 1, interferon-inducible protein p78 (mouse) | 2.90 | 1.54 |
| C10orf10 | 209183_s_at | AL136653 | chromosome 10 open reading frame 10 | 2.90 | 1.54 |
| ICAM1 | 202638_s_at | NM_000201 | intercellular adhesion molecule 1 (CD54), human rhinovirus receptor | 2.89 | 1.53 |
| ABCG1 | 204567_s_at | NM_004915 | ATP-binding cassette, sub-family G (WHITE), member 1 | 2.89 | 1.52 |
| MEGF6 | 226869_at | AI655611 | multiple EGF-like-domains 6 | 2.87 | 1.53 |
| LGALS7/LOC653499/LOC728910/LOC732032 | 206400_at | NM_002307 | lectin, galactoside-binding, soluble, 7 (galectin 7)///similar to Galectin-7 (Gal-7) (HKL-14) (PI7) (p53-induced protein 1) | 2.85 | 1.51 |
| SYNPO | 202796_at | NM_007286 | synaptopodin | 2.83 | 1.50 |
| KIAA1199 | 212942_s_at | AB033025 | KIAA1199 | 2.82 | 1.50 |
| SLC16A3 | 202856_s_at | NM_004207 | solute carrier family 16, member 3 (monocarboxylic acid transporter 4) | 2.79 | 1.48 |
| CTHRC1 | 225681_at | AA584310 | collagen triple helix repeat containing 1 | 2.77 | 1.47 |
| FLRT2 | 240259_at | AI188161 | Fibronectin leucine rich transmembrane protein 2 | 2.77 | 1.46 |
| RSAD2 | 213797_at | AI337069 | radical S-adenosyl methionine domain containing 2 | 2.76 | 1.45 |
| RSAD2 | 242625_at | AW189843 | radical S-adenosyl methionine domain containing 2 | 2.75 | 1.46 |
| DUSP6 | 208891_at | BC003143 | dual specificity phosphatase 6 | 2.75 | 1.46 |
| DUSP6 | 208893_s_at | BC005047 | dual specificity phosphatase 6 | 2.57 | 1.36 |
| PIK3IP1 | 221756_at | AL540260 | phosphoinositide-3-kinase interacting protein 1 | 2.73 | 1.45 |
| KLF7 | 204334_at | AA488672 | Kruppel-like factor 7 (ubiquitous) | 2.72 | 1.44 |
| SLC6A8 | 202219_at | NM_005629 | solute carrier family 6 (neurotransmitter transporter, creatine), member 8 | 2.70 | 1.43 |
| MGC5618 | 221477_s_at | BF575213 | hypothetical protein MGC5618 | 2.67 | 1.42 |
| FAM113B | 228298_at | BF056901 | family with sequence similarity 113, member B | 2.67 | 1.42 |
| RNASE4 | 213397_x_at | AI761728 | ribonuclease, RNase A family, 4 | 2.67 | 1.41 |
| ALDH1L2 | 231202_at | AI654224 | aldehyde dehydrogenase 1 family, member L2 | 2.65 | 1.40 |
| TCN1 | 205513_at | NM_001062 | transcobalamin I (vitamin B12 binding protein, R binder family) | 2.65 | 1.40 |
| COL17A1 | 204636_at | NM_000494 | collagen, type XVII, alpha 1 | 2.63 | 1.39 |
| MTSS1 | 203037_s_at | NM_014751 | metastasis suppressor 1 | 2.62 | 1.38 |
| RBP1 | 203423_at | NM_002899 | retinol binding protein 1, cellular | 2.57 | 1.36 |
| HTRA1 | 201185_at | NM_002775 | HtrA serine peptidase 1 | 2.55 | 1.35 |
| COL7A1 | 204136_at | NM_000094 | collagen, type VII, alpha 1 (epidermolysis bullosa, dystrophic, dominant and recessive) | 2.55 | 1.35 |
| OAS1 | 205552_s_at | NM_002534 | 2',5'-oligoadenylate synthetase 1, 40/46kDa | 2.54 | 1.34 |
| CRIP1 | 205081_at | NM_001311 | cysteine-rich protein 1 (intestinal) | 2.54 | 1.34 |
| G0S2 | 213524_s_at | NM_015714 | G0/G1switch 2 | 2.52 | 1.34 |
| SLC44A1 | 228486_at | AW165999 | solute carrier family 44, member 1 | 2.52 | 1.33 |
| PQLC3 | 225579_at | AL516202 | PQ loop repeat containing 3 | 2.51 | 1.33 |
| ISG20 | 204698_at | NM_002201 | interferon stimulated exonuclease gene 20kDa | 2.51 | 1.32 |
| SPAG4 | 219888_at | NM_003116 | sperm associated antigen 4 | 2.48 | 1.31 |
| STC1 | 204597_x_at | NM_003155 | stanniocalcin 1 | 2.47 | 1.30 |
| LAMP3 | 205569_at | NM_014398 | lysosomal-associated membrane protein 3 | 2.45 | 1.29 |
| LOXL2 | 202998_s_at | NM_002318 | lysyl oxidase-like 2 | 2.44 | 1.29 |
| AHNAK2 | 212992_at | AI935123 | AHNAK nucleoprotein 2 | 2.43 | 1.28 |
| IFITM1 | 201601_x_at | NM_003641 | interferon induced transmembrane protein 1 (9-27) | 2.42 | 1.27 |
| C1S | 208747_s_at | M18767 | complement component 1, s subcomponent | 2.38 | 1.25 |
| TIMP1 | 201666_at | NM_003254 | TIMP metallopeptidase inhibitor 1 | 2.34 | 1.23 |
| C1R | 212067_s_at | AL573058 | complement component 1, r subcomponent | 2.33 | 1.22 |
| ABCC3 | 208161_s_at | NM_020037 | ATP-binding cassette, sub-family C (CFTR/MRP), member 3 | 2.32 | 1.22 |
| SEMA4B | 234725_s_at | AK026133 | sema domain, immunoglobulin domain (Ig), transmembrane domain (TM) and short cytoplasmic domain, (semaphorin) 4B | 2.24 | 1.17 |
| TRIM22 | 213293_s_at | AA083478 | tripartite motif-containing 22 | 2.23 | 1.16 |
| OAS3 | 218400_at | NM_006187 | 2'-5'-oligoadenylate synthetase 3, 100kDa | 2.23 | 1.16 |
|  |  |  |  |  |  |
| **Genes downregulated in 3D versus 2D** | | |  |  |  |
| CYP4B1 | 210096_at | J02871 | cytochrome P450, family 4, subfamily B, polypeptide 1 | -19.33 | -4.29 |
| CTGF | 209101_at | M92934 | connective tissue growth factor | -14.42 | -3.85 |
| RPTN | 1553454_at | NM_152364 | repetin | -10.40 | -3.37 |
| VGLL1 | 215729_s_at | BE542323 | vestigial like 1 (Drosophila) | -8.89 | -3.16 |
| VGLL1 | 205487_s_at | NM_016267 | vestigial like 1 (Drosophila) | -2.76 | -1.47 |
| KRT4 | 213240_s_at | X07695 | keratin 4 | -7.58 | -2.93 |
| KRT4 | 214399_s_at | BF588953 | Keratin 4 | -4.83 | -2.25 |
| GRHL3 | 232116_at | AL137763 | grainyhead-like 3 (Drosophila) | -4.64 | -2.22 |
| KRT13 | 207935_s_at | NM_002274 | keratin 13 | -4.43 | -2.17 |
| HPGD | 203913_s_at | AL574184 | hydroxyprostaglandin dehydrogenase 15-(NAD) | -4.28 | -2.09 |
| HPGD | 203914_x_at | NM_000860 | hydroxyprostaglandin dehydrogenase 15-(NAD) | -4.13 | -2.05 |
| SCEL | 232056_at | AW470178 | sciellin | -4.24 | -2.08 |
| SCEL | 206884_s_at | NM_003843 | sciellin | -3.47 | -1.78 |
| RHOBTB3 | 216048_s_at | AK023621 | Rho-related BTB domain containing 3 | -4.16 | -2.06 |
| RHOBTB3 | 202975_s_at | N21138 | Rho-related BTB domain containing 3 | -4.03 | -2.01 |
| RHOBTB3 | 225202_at | BE620739 | Rho-related BTB domain containing 3 | -3.36 | -1.76 |
| RHOBTB3 | 202976_s_at | NM_014899 | Rho-related BTB domain containing 3 | -3.06 | -1.62 |
| SLC20A1 | 230494_at | AI671885 | Solute carrier family 20 (phosphate transporter), member 1 | -4.03 | -1.99 |
| KRT24 | 220267_at | NM_019016 | keratin 24 | -3.80 | -1.93 |
| CLDN4 | 201428_at | NM_001305 | claudin 4 | -3.70 | -1.90 |
| CRNN | 220090_at | NM_016190 | cornulin | -3.60 | -1.84 |
| CYR61 | 210764_s_at | AF003114 | cysteine-rich, angiogenic inducer, 61 | -3.49 | -1.80 |
| CYR61 | 201289_at | NM_001554 | cysteine-rich, angiogenic inducer, 61 | -3.17 | -1.67 |
| DUSP1 | 201041_s_at | NM_004417 | dual specificity phosphatase 1 | -3.42 | -1.77 |
| CTH | 217127_at | AL354872 | cystathionase (cystathionine gamma-lyase) | -3.41 | -1.77 |
| CTH | 206085_s_at | NM_001902 | cystathionase (cystathionine gamma-lyase) | -3.13 | -1.65 |
| IL6 | 205207_at | NM_000600 | interleukin 6 (interferon, beta 2) | -3.41 | -1.77 |
| ARG2 | 203946_s_at | U75667 | arginase, type II | -3.34 | -1.74 |
| CCNL1 | 1555827_at | AY034790 | Cyclin L1 | -3.17 | -1.66 |
| MAP3K8 | 235421_at | AV713062 | CDNA clone IMAGE:4689481///Mitogen-activated protein kinase kinase kinase 8 | -3.15 | -1.65 |
| DUSP10 | 215501_s_at | AK022513 | dual specificity phosphatase 10 | -3.14 | -1.65 |
| DUSP10 | 221563_at | N36770 | dual specificity phosphatase 10 | -3.11 | -1.64 |
| KRT80 | 231849_at | AL162069 | keratin 80 | -3.09 | -1.62 |
| FOXA1 | 204667_at | NM_004496 | forkhead box A1 | -3.06 | -1.62 |
| ZNF750 | 219995_s_at | NM_024702 | zinc finger protein 750 | -3.05 | -1.61 |
| MUC20 | 230043_at | AI168678 | mucin 20, cell surface associated | -3.01 | -1.59 |
| KIAA0114 | 224870_at | BG255416 | KIAA0114 | -2.98 | -1.57 |
| ANKRD22 | 238439_at | AI925518 | ankyrin repeat domain 22 | -2.86 | -1.51 |
| BCL10 | 1557257_at | AA994334 | B-cell CLL/lymphoma 10 | -2.83 | -1.50 |
| CYP4F3 | 206515_at | NM_000896 | cytochrome P450, family 4, subfamily F, polypeptide 3 | -2.82 | -1.50 |
| LMO7 | 242722_at | AA100793 | LIM domain 7 | -2.71 | -1.44 |
| MTHFD2L | 238762_at | AA702016 | methylenetetrahydrofolate dehydrogenase (NADP+ dependent) 2-like | -2.68 | -1.42 |
| GCNT3 | 219508_at | NM_004751 | glucosaminyl (N-acetyl) transferase 3, mucin type | -2.66 | -1.42 |
| UPK1B | 210065_s_at | AB002155 | uroplakin 1B | -2.66 | -1.41 |
| NEFL | 221916_at | BF055311 | neurofilament, light polypeptide 68kDa | -2.51 | -1.33 |
| PLEKHA7 | 228450_at | AA758861 | pleckstrin homology domain containing, family A member 7 | -2.28 | -1.19 |

^a^Listing of up- and downregulated genes according to SAM.
