# Supplementary material for: Genome-Wide Gene Expression Analysis in Cancer Cells Reveals 3D Growth to Affect ECM and Processes Associated with Cell Adhesion but Not DNA Repair
Source: PLoS One. 2012 Apr 11;7(4):e34279. doi: 10.1371/journal.pone.0034279 (PMC3324525; doi:10.1371/journal.pone.0034279)
Supplement: Table S3 — Gene Ontology Analysis, Cellular Component. The overlap is marked in italic/bold letters. (DOCX) [file pone.0034279.s003.docx]

**Table S3.** Gene Ontology Analysis, Cellular Component. The overlap is marked in italic/bold letters.

| **A549** | **3D vs 2D** |  |  |  |
| --- | --- | --- | --- | --- |
|  | ID | Name | P-value | Term in Query |
| ***1*** | ***GO:0044421*** | ***extracellular region part*** | ***1,58E-20*** | [***61***](http://toppgene.cchmc.org/showQueryTerms.jsp?userdata_id=2b040fb3-d381-4da1-b398-e512ce150561&feature=goc&row=0) |
| ***2*** | ***GO:0005615*** | ***extracellular space*** | ***1,41E-16*** | [***49***](http://toppgene.cchmc.org/showQueryTerms.jsp?userdata_id=2b040fb3-d381-4da1-b398-e512ce150561&feature=goc&row=1) |
| ***3*** | ***GO:0031012*** | ***extracellular matrix*** | ***2,77E-09*** | [***26***](http://toppgene.cchmc.org/showQueryTerms.jsp?userdata_id=2b040fb3-d381-4da1-b398-e512ce150561&feature=goc&row=2) |
| **4** | GO:0031983 | vesicle lumen | 2,53E-07 | [10](http://toppgene.cchmc.org/showQueryTerms.jsp?userdata_id=2b040fb3-d381-4da1-b398-e512ce150561&feature=goc&row=3) |
| **5** | GO:0031093 | platelet alpha granule lumen | 1,86E-06 | [9](http://toppgene.cchmc.org/showQueryTerms.jsp?userdata_id=2b040fb3-d381-4da1-b398-e512ce150561&feature=goc&row=4) |
| **6** | GO:0034774 | secretory granule lumen | 2,27E-06 | [9](http://toppgene.cchmc.org/showQueryTerms.jsp?userdata_id=2b040fb3-d381-4da1-b398-e512ce150561&feature=goc&row=5) |
| **7** | GO:0060205 | cytoplasmic membrane-bounded vesicle lumen | 2,74E-06 | [9](http://toppgene.cchmc.org/showQueryTerms.jsp?userdata_id=2b040fb3-d381-4da1-b398-e512ce150561&feature=goc&row=6) |
| **8** | GO:0005577 | fibrinogen complex | 4,75E-06 | [5](http://toppgene.cchmc.org/showQueryTerms.jsp?userdata_id=2b040fb3-d381-4da1-b398-e512ce150561&feature=goc&row=7) |
| ***9*** | ***GO:0005578*** | ***proteinaceous extracellular matrix*** | ***2,13E-05*** | [***19***](http://toppgene.cchmc.org/showQueryTerms.jsp?userdata_id=2b040fb3-d381-4da1-b398-e512ce150561&feature=goc&row=8) |
| **10** | GO:0031091 | platelet alpha granule | 2,71E-05 | [9](http://toppgene.cchmc.org/showQueryTerms.jsp?userdata_id=2b040fb3-d381-4da1-b398-e512ce150561&feature=goc&row=9) |
| ***11*** | ***GO:0044420*** | ***extracellular matrix part*** | ***4,08E-05*** | [***12***](http://toppgene.cchmc.org/showQueryTerms.jsp?userdata_id=2b040fb3-d381-4da1-b398-e512ce150561&feature=goc&row=10) |
| ***12*** | ***GO:0005604*** | ***basement membrane*** | ***5,99E-05*** | [***10***](http://toppgene.cchmc.org/showQueryTerms.jsp?userdata_id=2b040fb3-d381-4da1-b398-e512ce150561&feature=goc&row=11) |
| **13** | GO:0016323 | basolateral plasma membrane | 6,89E-04 | [15](http://toppgene.cchmc.org/showQueryTerms.jsp?userdata_id=2b040fb3-d381-4da1-b398-e512ce150561&feature=goc&row=12) |
| **14** | GO:0030141 | stored secretory granule | 1,32E-03 | [14](http://toppgene.cchmc.org/showQueryTerms.jsp?userdata_id=2b040fb3-d381-4da1-b398-e512ce150561&feature=goc&row=13) |

| **UT-SCC15** | **3D vs 2D** |  |  |  |
| --- | --- | --- | --- | --- |
|  | ID | Name | P-value | Term in Query |
| ***1*** | ***GO:0044421*** | ***extracellular region part*** | ***3,44E-08*** | [***29***](http://toppgene.cchmc.org/showQueryTerms.jsp?userdata_id=a574ac4c-be1e-4bdb-90e7-2ef736f2fe82&feature=goc&row=0) |
| ***2*** | ***GO:0031012*** | ***extracellular matrix*** | ***2,06E-06*** | [***16***](http://toppgene.cchmc.org/showQueryTerms.jsp?userdata_id=a574ac4c-be1e-4bdb-90e7-2ef736f2fe82&feature=goc&row=1) |
| ***3*** | ***GO:0005578*** | ***proteinaceous extracellular matrix*** | ***1,26E-05*** | [***14***](http://toppgene.cchmc.org/showQueryTerms.jsp?userdata_id=a574ac4c-be1e-4bdb-90e7-2ef736f2fe82&feature=goc&row=2) |
| ***4*** | ***GO:0005615*** | ***extracellular space*** | ***8,56E-04*** | [***19***](http://toppgene.cchmc.org/showQueryTerms.jsp?userdata_id=a574ac4c-be1e-4bdb-90e7-2ef736f2fe82&feature=goc&row=3) |
| ***5*** | ***GO:0005604*** | ***basement membrane*** | ***5,91E-03*** | [***6***](http://toppgene.cchmc.org/showQueryTerms.jsp?userdata_id=a574ac4c-be1e-4bdb-90e7-2ef736f2fe82&feature=goc&row=4) |
| ***6*** | ***GO:0044420*** | ***extracellular matrix part*** | ***6,25E-03*** | [***7***](http://toppgene.cchmc.org/showQueryTerms.jsp?userdata_id=a574ac4c-be1e-4bdb-90e7-2ef736f2fe82&feature=goc&row=5) |
| **7** | GO:0009925 | basal plasma membrane | 1,11E-02 | [4](http://toppgene.cchmc.org/showQueryTerms.jsp?userdata_id=a574ac4c-be1e-4bdb-90e7-2ef736f2fe82&feature=goc&row=6) |
| **8** | GO:0045178 | basal part of cell | 1,99E-02 | [4](http://toppgene.cchmc.org/showQueryTerms.jsp?userdata_id=a574ac4c-be1e-4bdb-90e7-2ef736f2fe82&feature=goc&row=7) |
| **9** | GO:0005610 | laminin-5 complex | 4,90E-02 | [2](http://toppgene.cchmc.org/showQueryTerms.jsp?userdata_id=a574ac4c-be1e-4bdb-90e7-2ef736f2fe82&feature=goc&row=8) |
