# Supplementary material for: Genome-Wide Gene Expression Analysis in Cancer Cells Reveals 3D Growth to Affect ECM and Processes Associated with Cell Adhesion but Not DNA Repair
Source: PLoS One. 2012 Apr 11;7(4):e34279. doi: 10.1371/journal.pone.0034279 (PMC3324525; doi:10.1371/journal.pone.0034279)
Supplement: Table S4 — Gene Ontology Analysis, Biological Process. The overlap is marked in italic/bold letters. (DOCX) [file pone.0034279.s004.docx]

**Table S4.** Gene Ontology Analysis, Biological Process. The overlap is marked in italic/bold letters.

| **A549 3D vs 2D** | | **SAM** |  |  |
| --- | --- | --- | --- | --- |
|  | ID | Name | P-value | Term in Query |
| **1** | GO:0009611 | response to wounding | 2,83E-17 | [56](http://toppgene.cchmc.org/showQueryTerms.jsp?userdata_id=2b040fb3-d381-4da1-b398-e512ce150561&feature=gop&row=0) |
| **2** | GO:0042060 | wound healing | 1,05E-11 | [37](http://toppgene.cchmc.org/showQueryTerms.jsp?userdata_id=2b040fb3-d381-4da1-b398-e512ce150561&feature=gop&row=1) |
| **3** | GO:0050878 | regulation of body fluid levels | 3,37E-09 | [33](http://toppgene.cchmc.org/showQueryTerms.jsp?userdata_id=2b040fb3-d381-4da1-b398-e512ce150561&feature=gop&row=2) |
| **4** | GO:0007596 | blood coagulation | 2,42E-07 | [28](http://toppgene.cchmc.org/showQueryTerms.jsp?userdata_id=2b040fb3-d381-4da1-b398-e512ce150561&feature=gop&row=3) |
| **5** | GO:0050817 | coagulation | 2,77E-07 | [28](http://toppgene.cchmc.org/showQueryTerms.jsp?userdata_id=2b040fb3-d381-4da1-b398-e512ce150561&feature=gop&row=4) |
| **6** | GO:0070887 | cellular response to chemical stimulus | 2,83E-07 | [36](http://toppgene.cchmc.org/showQueryTerms.jsp?userdata_id=2b040fb3-d381-4da1-b398-e512ce150561&feature=gop&row=5) |
| **7** | GO:0007599 | hemostasis | 2,90E-07 | [28](http://toppgene.cchmc.org/showQueryTerms.jsp?userdata_id=2b040fb3-d381-4da1-b398-e512ce150561&feature=gop&row=6) |
| **8** | GO:0010035 | response to inorganic substance | 8,81E-07 | [21](http://toppgene.cchmc.org/showQueryTerms.jsp?userdata_id=2b040fb3-d381-4da1-b398-e512ce150561&feature=gop&row=7) |
| **9** | GO:0051241 | negative regulation of multicellular organismal process | 1,37E-06 | [20](http://toppgene.cchmc.org/showQueryTerms.jsp?userdata_id=2b040fb3-d381-4da1-b398-e512ce150561&feature=gop&row=8) |
| **10** | GO:0009605 | response to external stimulus | 3,43E-06 | [41](http://toppgene.cchmc.org/showQueryTerms.jsp?userdata_id=2b040fb3-d381-4da1-b398-e512ce150561&feature=gop&row=9) |
| **11** | GO:0072359 | circulatory system development | 3,78E-06 | [29](http://toppgene.cchmc.org/showQueryTerms.jsp?userdata_id=2b040fb3-d381-4da1-b398-e512ce150561&feature=gop&row=10) |
| **12** | GO:0072358 | cardiovascular system development | 3,78E-06 | [29](http://toppgene.cchmc.org/showQueryTerms.jsp?userdata_id=2b040fb3-d381-4da1-b398-e512ce150561&feature=gop&row=11) |
| **13** | GO:0032101 | regulation of response to external stimulus | 4,22E-06 | [19](http://toppgene.cchmc.org/showQueryTerms.jsp?userdata_id=2b040fb3-d381-4da1-b398-e512ce150561&feature=gop&row=12) |
| **14** | GO:0046903 | secretion | 8,40E-06 | [32](http://toppgene.cchmc.org/showQueryTerms.jsp?userdata_id=2b040fb3-d381-4da1-b398-e512ce150561&feature=gop&row=13) |
| **15** | GO:0051239 | regulation of multicellular organismal process | 8,94E-06 | [47](http://toppgene.cchmc.org/showQueryTerms.jsp?userdata_id=2b040fb3-d381-4da1-b398-e512ce150561&feature=gop&row=14) |
| **16** | GO:0006928 | cellular component movement | 1,28E-05 | [33](http://toppgene.cchmc.org/showQueryTerms.jsp?userdata_id=2b040fb3-d381-4da1-b398-e512ce150561&feature=gop&row=15) |
| **17** | GO:0002576 | platelet degranulation | 1,63E-05 | [11](http://toppgene.cchmc.org/showQueryTerms.jsp?userdata_id=2b040fb3-d381-4da1-b398-e512ce150561&feature=gop&row=16) |
| **18** | GO:0050818 | regulation of coagulation | 2,91E-05 | [9](http://toppgene.cchmc.org/showQueryTerms.jsp?userdata_id=2b040fb3-d381-4da1-b398-e512ce150561&feature=gop&row=17) |
| **19** | GO:0016477 | cell migration | 3,21E-05 | [29](http://toppgene.cchmc.org/showQueryTerms.jsp?userdata_id=2b040fb3-d381-4da1-b398-e512ce150561&feature=gop&row=18) |
| **20** | GO:0050819 | negative regulation of coagulation | 3,94E-05 | [8](http://toppgene.cchmc.org/showQueryTerms.jsp?userdata_id=2b040fb3-d381-4da1-b398-e512ce150561&feature=gop&row=19) |
| **21** | GO:0006954 | inflammatory response | 5,56E-05 | [22](http://toppgene.cchmc.org/showQueryTerms.jsp?userdata_id=2b040fb3-d381-4da1-b398-e512ce150561&feature=gop&row=20) |
| **22** | GO:0090066 | regulation of anatomical structure size | 7,52E-05 | [25](http://toppgene.cchmc.org/showQueryTerms.jsp?userdata_id=2b040fb3-d381-4da1-b398-e512ce150561&feature=gop&row=21) |
| *23* | ***GO:0010033*** | ***response to organic substance*** | ***8,25E-05*** | [***40***](http://toppgene.cchmc.org/showQueryTerms.jsp?userdata_id=2b040fb3-d381-4da1-b398-e512ce150561&feature=gop&row=22) |
| **24** | GO:0008283 | cell proliferation | 8,65E-05 | [44](http://toppgene.cchmc.org/showQueryTerms.jsp?userdata_id=2b040fb3-d381-4da1-b398-e512ce150561&feature=gop&row=23) |
| **25** | GO:0048646 | anatomical structure formation involved in morphogenesis | 9,73E-05 | [27](http://toppgene.cchmc.org/showQueryTerms.jsp?userdata_id=2b040fb3-d381-4da1-b398-e512ce150561&feature=gop&row=24) |
| *26* | ***GO:0007155*** | ***cell adhesion*** | ***1,02E-04*** | [***33***](http://toppgene.cchmc.org/showQueryTerms.jsp?userdata_id=2b040fb3-d381-4da1-b398-e512ce150561&feature=gop&row=25) |
| *27* | ***GO:0022610*** | ***biological adhesion*** | ***1,02E-04*** | [***33***](http://toppgene.cchmc.org/showQueryTerms.jsp?userdata_id=2b040fb3-d381-4da1-b398-e512ce150561&feature=gop&row=26) |
| **28** | GO:0001568 | blood vessel development | 1,03E-04 | [21](http://toppgene.cchmc.org/showQueryTerms.jsp?userdata_id=2b040fb3-d381-4da1-b398-e512ce150561&feature=gop&row=27) |
| **29** | GO:0051674 | localization of cell | 1,24E-04 | [29](http://toppgene.cchmc.org/showQueryTerms.jsp?userdata_id=2b040fb3-d381-4da1-b398-e512ce150561&feature=gop&row=28) |
| **30** | GO:0048870 | cell motility | 1,24E-04 | [29](http://toppgene.cchmc.org/showQueryTerms.jsp?userdata_id=2b040fb3-d381-4da1-b398-e512ce150561&feature=gop&row=29) |
| **31** | GO:0030193 | regulation of blood coagulation | 1,75E-04 | [8](http://toppgene.cchmc.org/showQueryTerms.jsp?userdata_id=2b040fb3-d381-4da1-b398-e512ce150561&feature=gop&row=30) |
| **32** | GO:0001944 | vasculature development | 1,79E-04 | [21](http://toppgene.cchmc.org/showQueryTerms.jsp?userdata_id=2b040fb3-d381-4da1-b398-e512ce150561&feature=gop&row=31) |
| *33* | ***GO:0006952*** | ***defense response*** | ***1,82E-04*** | [***33***](http://toppgene.cchmc.org/showQueryTerms.jsp?userdata_id=2b040fb3-d381-4da1-b398-e512ce150561&feature=gop&row=32) |
| **34** | GO:0048514 | blood vessel morphogenesis | 2,00E-04 | [19](http://toppgene.cchmc.org/showQueryTerms.jsp?userdata_id=2b040fb3-d381-4da1-b398-e512ce150561&feature=gop&row=33) |
| *35* | ***GO:0002376*** | ***immune system process*** | ***2,02E-04*** | [***44***](http://toppgene.cchmc.org/showQueryTerms.jsp?userdata_id=2b040fb3-d381-4da1-b398-e512ce150561&feature=gop&row=34) |
| **36** | GO:0048545 | response to steroid hormone stimulus | 2,23E-04 | [18](http://toppgene.cchmc.org/showQueryTerms.jsp?userdata_id=2b040fb3-d381-4da1-b398-e512ce150561&feature=gop&row=35) |
| **37** | GO:0030168 | platelet activation | 2,94E-04 | [16](http://toppgene.cchmc.org/showQueryTerms.jsp?userdata_id=2b040fb3-d381-4da1-b398-e512ce150561&feature=gop&row=36) |
| *38* | ***GO:0009888*** | ***tissue development*** | ***3,21E-04*** | [***36***](http://toppgene.cchmc.org/showQueryTerms.jsp?userdata_id=2b040fb3-d381-4da1-b398-e512ce150561&feature=gop&row=37) |
| **39** | GO:0030195 | negative regulation of blood coagulation | 3,63E-04 | [7](http://toppgene.cchmc.org/showQueryTerms.jsp?userdata_id=2b040fb3-d381-4da1-b398-e512ce150561&feature=gop&row=38) |
| **40** | GO:0048583 | regulation of response to stimulus | 4,26E-04 | [31](http://toppgene.cchmc.org/showQueryTerms.jsp?userdata_id=2b040fb3-d381-4da1-b398-e512ce150561&feature=gop&row=39) |
| **41** | GO:0009719 | response to endogenous stimulus | 4,45E-04 | [28](http://toppgene.cchmc.org/showQueryTerms.jsp?userdata_id=2b040fb3-d381-4da1-b398-e512ce150561&feature=gop&row=40) |
| **42** | GO:0061041 | regulation of wound healing | 5,99E-04 | [8](http://toppgene.cchmc.org/showQueryTerms.jsp?userdata_id=2b040fb3-d381-4da1-b398-e512ce150561&feature=gop&row=41) |
| **43** | GO:0010038 | response to metal ion | 6,50E-04 | [14](http://toppgene.cchmc.org/showQueryTerms.jsp?userdata_id=2b040fb3-d381-4da1-b398-e512ce150561&feature=gop&row=42) |
| **44** | GO:0042730 | fibrinolysis | 7,10E-04 | [6](http://toppgene.cchmc.org/showQueryTerms.jsp?userdata_id=2b040fb3-d381-4da1-b398-e512ce150561&feature=gop&row=43) |
| **45** | GO:0032535 | regulation of cellular component size | 8,34E-04 | [21](http://toppgene.cchmc.org/showQueryTerms.jsp?userdata_id=2b040fb3-d381-4da1-b398-e512ce150561&feature=gop&row=44) |
| **46** | GO:0040011 | locomotion | 9,60E-04 | [35](http://toppgene.cchmc.org/showQueryTerms.jsp?userdata_id=2b040fb3-d381-4da1-b398-e512ce150561&feature=gop&row=45) |
| **47** | GO:0040007 | growth | 1,37E-03 | [27](http://toppgene.cchmc.org/showQueryTerms.jsp?userdata_id=2b040fb3-d381-4da1-b398-e512ce150561&feature=gop&row=46) |
| **48** | GO:0030194 | positive regulation of blood coagulation | 1,63E-03 | [5](http://toppgene.cchmc.org/showQueryTerms.jsp?userdata_id=2b040fb3-d381-4da1-b398-e512ce150561&feature=gop&row=47) |
| **49** | GO:0001775 | cell activation | 1,75E-03 | [27](http://toppgene.cchmc.org/showQueryTerms.jsp?userdata_id=2b040fb3-d381-4da1-b398-e512ce150561&feature=gop&row=48) |
| **50** | GO:2000026 | regulation of multicellular organismal development | 4,38E-03 | [29](http://toppgene.cchmc.org/showQueryTerms.jsp?userdata_id=2b040fb3-d381-4da1-b398-e512ce150561&feature=gop&row=49) |
| **51** | GO:0050820 | positive regulation of coagulation | 4,88E-03 | [5](http://toppgene.cchmc.org/showQueryTerms.jsp?userdata_id=2b040fb3-d381-4da1-b398-e512ce150561&feature=gop&row=50) |
| **52** | GO:0008285 | negative regulation of cell proliferation | 5,72E-03 | [20](http://toppgene.cchmc.org/showQueryTerms.jsp?userdata_id=2b040fb3-d381-4da1-b398-e512ce150561&feature=gop&row=51) |
| **53** | GO:0051240 | positive regulation of multicellular organismal process | 6,52E-03 | [18](http://toppgene.cchmc.org/showQueryTerms.jsp?userdata_id=2b040fb3-d381-4da1-b398-e512ce150561&feature=gop&row=52) |
| **54** | GO:0032940 | secretion by cell | 7,00E-03 | [24](http://toppgene.cchmc.org/showQueryTerms.jsp?userdata_id=2b040fb3-d381-4da1-b398-e512ce150561&feature=gop&row=53) |
| **55** | GO:0001525 | angiogenesis | 8,63E-03 | [15](http://toppgene.cchmc.org/showQueryTerms.jsp?userdata_id=2b040fb3-d381-4da1-b398-e512ce150561&feature=gop&row=54) |
| **56** | GO:0009725 | response to hormone stimulus | 8,67E-03 | [24](http://toppgene.cchmc.org/showQueryTerms.jsp?userdata_id=2b040fb3-d381-4da1-b398-e512ce150561&feature=gop&row=55) |
| **57** | GO:0051592 | response to calcium ion | 1,05E-02 | [8](http://toppgene.cchmc.org/showQueryTerms.jsp?userdata_id=2b040fb3-d381-4da1-b398-e512ce150561&feature=gop&row=56) |
| **58** | GO:0042127 | regulation of cell proliferation | 1,15E-02 | [32](http://toppgene.cchmc.org/showQueryTerms.jsp?userdata_id=2b040fb3-d381-4da1-b398-e512ce150561&feature=gop&row=57) |
| **59** | GO:0051918 | negative regulation of fibrinolysis | 1,40E-02 | [4](http://toppgene.cchmc.org/showQueryTerms.jsp?userdata_id=2b040fb3-d381-4da1-b398-e512ce150561&feature=gop&row=58) |
| **60** | GO:0002526 | acute inflammatory response | 1,40E-02 | [9](http://toppgene.cchmc.org/showQueryTerms.jsp?userdata_id=2b040fb3-d381-4da1-b398-e512ce150561&feature=gop&row=59) |
| **61** | GO:0050793 | regulation of developmental process | 1,83E-02 | [31](http://toppgene.cchmc.org/showQueryTerms.jsp?userdata_id=2b040fb3-d381-4da1-b398-e512ce150561&feature=gop&row=60) |
| **62** | GO:0016049 | cell growth | 2,26E-02 | [16](http://toppgene.cchmc.org/showQueryTerms.jsp?userdata_id=2b040fb3-d381-4da1-b398-e512ce150561&feature=gop&row=61) |
| **63** | GO:0072376 | protein activation cascade | 3,05E-02 | [8](http://toppgene.cchmc.org/showQueryTerms.jsp?userdata_id=2b040fb3-d381-4da1-b398-e512ce150561&feature=gop&row=62) |
| **64** | GO:0050900 | leukocyte migration | 3,69E-02 | [12](http://toppgene.cchmc.org/showQueryTerms.jsp?userdata_id=2b040fb3-d381-4da1-b398-e512ce150561&feature=gop&row=63) |
| **65** | GO:0007167 | enzyme linked receptor protein signaling pathway | 3,84E-02 | [26](http://toppgene.cchmc.org/showQueryTerms.jsp?userdata_id=2b040fb3-d381-4da1-b398-e512ce150561&feature=gop&row=64) |
| **66** | GO:0008361 | regulation of cell size | 4,45E-02 | [16](http://toppgene.cchmc.org/showQueryTerms.jsp?userdata_id=2b040fb3-d381-4da1-b398-e512ce150561&feature=gop&row=65) |
| **67** | GO:0051917 | regulation of fibrinolysis | 4,61E-02 | [4](http://toppgene.cchmc.org/showQueryTerms.jsp?userdata_id=2b040fb3-d381-4da1-b398-e512ce150561&feature=gop&row=66) |

| **UT-SCC15 3D vs 2D** | | **SAM** |  |  |
| --- | --- | --- | --- | --- |
|  | ID | Name | P-value | Term in Query |
| **1** | GO:0060337 | type I interferon-mediated signaling pathway | 1,62E-06 | [9](http://toppgene.cchmc.org/showQueryTerms.jsp?userdata_id=a574ac4c-be1e-4bdb-90e7-2ef736f2fe82&feature=gop&row=0) |
| *2* | ***GO:0002376*** | ***immune system process*** | ***4,06E-05*** | [***30***](http://toppgene.cchmc.org/showQueryTerms.jsp?userdata_id=a574ac4c-be1e-4bdb-90e7-2ef736f2fe82&feature=gop&row=1) |
| *3* | ***GO:0007155*** | ***cell adhesion*** | ***1,91E-04*** | [***22***](http://toppgene.cchmc.org/showQueryTerms.jsp?userdata_id=a574ac4c-be1e-4bdb-90e7-2ef736f2fe82&feature=gop&row=2) |
| *4* | ***GO:0022610*** | ***biological adhesion*** | ***1,91E-04*** | [***22***](http://toppgene.cchmc.org/showQueryTerms.jsp?userdata_id=a574ac4c-be1e-4bdb-90e7-2ef736f2fe82&feature=gop&row=3) |
| **5** | GO:0006955 | immune response | 8,83E-04 | [21](http://toppgene.cchmc.org/showQueryTerms.jsp?userdata_id=a574ac4c-be1e-4bdb-90e7-2ef736f2fe82&feature=gop&row=4) |
| **6** | GO:0051707 | response to other organism | 1,00E-03 | [15](http://toppgene.cchmc.org/showQueryTerms.jsp?userdata_id=a574ac4c-be1e-4bdb-90e7-2ef736f2fe82&feature=gop&row=5) |
| **7** | GO:0019221 | cytokine-mediated signaling pathway | 1,00E-03 | [11](http://toppgene.cchmc.org/showQueryTerms.jsp?userdata_id=a574ac4c-be1e-4bdb-90e7-2ef736f2fe82&feature=gop&row=6) |
| **8** | GO:0008544 | epidermis development | 1,93E-03 | [11](http://toppgene.cchmc.org/showQueryTerms.jsp?userdata_id=a574ac4c-be1e-4bdb-90e7-2ef736f2fe82&feature=gop&row=7) |
| *9* | ***GO:0009888*** | ***tissue development*** | ***4,85E-03*** | [***22***](http://toppgene.cchmc.org/showQueryTerms.jsp?userdata_id=a574ac4c-be1e-4bdb-90e7-2ef736f2fe82&feature=gop&row=8) |
| *10* | ***GO:0006952*** | ***defense response*** | ***5,02E-03*** | [***20***](http://toppgene.cchmc.org/showQueryTerms.jsp?userdata_id=a574ac4c-be1e-4bdb-90e7-2ef736f2fe82&feature=gop&row=9) |
| **11** | GO:0052548 | regulation of endopeptidase activity | 8,52E-03 | [10](http://toppgene.cchmc.org/showQueryTerms.jsp?userdata_id=a574ac4c-be1e-4bdb-90e7-2ef736f2fe82&feature=gop&row=10) |
| **12** | GO:0009607 | response to biotic stimulus | 1,24E-02 | [15](http://toppgene.cchmc.org/showQueryTerms.jsp?userdata_id=a574ac4c-be1e-4bdb-90e7-2ef736f2fe82&feature=gop&row=11) |
| **13** | GO:0009615 | response to virus | 1,31E-02 | [9](http://toppgene.cchmc.org/showQueryTerms.jsp?userdata_id=a574ac4c-be1e-4bdb-90e7-2ef736f2fe82&feature=gop&row=12) |
| **14** | GO:0052547 | regulation of peptidase activity | 1,32E-02 | [10](http://toppgene.cchmc.org/showQueryTerms.jsp?userdata_id=a574ac4c-be1e-4bdb-90e7-2ef736f2fe82&feature=gop&row=13) |
| **15** | GO:0002237 | response to molecule of bacterial origin | 2,41E-02 | [8](http://toppgene.cchmc.org/showQueryTerms.jsp?userdata_id=a574ac4c-be1e-4bdb-90e7-2ef736f2fe82&feature=gop&row=14) |
| **16** | GO:0051704 | multi-organism process | 2,84E-02 | [19](http://toppgene.cchmc.org/showQueryTerms.jsp?userdata_id=a574ac4c-be1e-4bdb-90e7-2ef736f2fe82&feature=gop&row=15) |
| **17** | GO:0031960 | response to corticosteroid stimulus | 3,17E-02 | [7](http://toppgene.cchmc.org/showQueryTerms.jsp?userdata_id=a574ac4c-be1e-4bdb-90e7-2ef736f2fe82&feature=gop&row=16) |
| *18* | ***GO:0010033*** | ***response to organic substance*** | ***3,19E-02*** | [***22***](http://toppgene.cchmc.org/showQueryTerms.jsp?userdata_id=a574ac4c-be1e-4bdb-90e7-2ef736f2fe82&feature=gop&row=17) |
| **19** | GO:0002682 | regulation of immune system process | 4,39E-02 | [16](http://toppgene.cchmc.org/showQueryTerms.jsp?userdata_id=a574ac4c-be1e-4bdb-90e7-2ef736f2fe82&feature=gop&row=18) |
